# Exploring phytochemical, antioxidant, and antimicrobial properties of *Plumeria pudica* Jacq. leaves

**DOI:** 10.1038/s41598-024-83980-6

**Published:** 2025-01-02

**Authors:** Kavan Shukla, Kunal N. Odedra, B. A. Jadeja

**Affiliations:** Department of Botany, M.D. Science College, Porbandar, Gujarat 360575 India

**Keywords:** *Plumeria pudica*, Medicinal plants, Phytochemicals, Phenolics, Antioxidant potential, And antimicrobial potential, Biochemistry, Plant sciences

## Abstract

Since the emergence of the coronavirus disease, there has been a notable surge in demand for herbal remedies with minimal or no adverse effects. Notably, existing vaccines and medications employed in its treatment have exhibited significant side effects, some of which have proven fatal. Consequently, there is an increasing focus on pharmacological research aimed at identifying optimal solutions to this challenge. This shift entails exploring organic alternatives to traditional medicines, involving the extraction of superior phytochemicals from plants for enhanced biomedical applications in treating various diseases and conditions. To evaluate the qualitative phytochemicals and the quantity of these phytochemicals present in the leaf extracts of the medicinally important plant *Plumeria pudica* Jacq. Also, the antioxidant property estimation and the study of the antimicrobial properties of the plant have been done in this research. The qualitative phytochemical analysis was done to evaluate the presence of various phytochemicals and to quantify these phytochemicals total content estimation of them was done. Also, phytochemical analysis was further enriched by LCMS-QTOF analysis for the presence of compounds. The determination of the antioxidant potential of the leaves was done by two assays, the reducing power assay and the DPPH(2,2-diphenyl-1-picrylhydrazyl) assay. With that the antimicrobial properties of the leaves were also put to test against four bacterial strains namely, *Kocuria rhizophila, Pseudomonas aeruginosa, Klebsiella pneumonia*, and *E. coli*. The results of the phytochemical evaluation indicated that both IPA and hydroalcoholic extracts exhibited a superior phytochemical composition, emphasizing the higher extractive potential of IPA compared to the non-polar petroleum ether extract. The quantitative analysis revealed the predominance of IPA extract as the quantity of phenols (101 mg GAE/g dry-weight of plant extract), flavonoids (402.2 mg QE/g dry-weight of plant extract), carbohydrates (336 mg GLU/g dry-weight of plant extract), and proteins (164 mg BSAE/g dry-weight of plant extract) were highest in the IPA extract. LC–MS QTOF analysis demonstrated the presence of significant phytocompounds in all leaf extracts that have pharmacological applications. Moreover, in antioxidant assays, the IPA extract showed the highest DPPH scavenging activity (66.85% of inhibition), with an IC_50_ value of 33.54 µg/mL, and the IPA extract exhibited the highest reducing power (1.5 absorbance), signifying robust antioxidant activity. Furthermore, the antimicrobial evaluation revealed that the aqueous and hydroalcoholic extracts displayed larger zones of inhibition compared to the other leaf extracts. And, during the antimicrobial activity interestingly most susceptibility was shown by *Klebsiella pneumonia*. This study concludes that the diverse extracts of *P. pudica* leaves possess remarkable phytoconstituent properties both qualitatively and quantitatively, suggesting their rich bioactive compound content and potential as novel sources for therapeutic applications.

## Introduction

Plants have long been revered for their medicinal properties, which contribute to community health by providing a rich source of secondary metabolites. Different plant parts, such as bark, leaves, fruits, flowers, and roots, contain bioactive compounds that offer therapeutic benefits^1^. Phytochemicals are bioactive substances produced through the secondary metabolism of plants, and they offer significant health benefits for humans. These phytochemicals and their presence with the estimations of their quantity can help researchers to identify the plant’s biochemical richness as these biochemicals denote many pharmacological applications. Among the most well-known phytochemicals are phenolic compounds, tannins, saponins, carotenoids, coumarins, tocopherols, terpenes, isothiocyanates, sulfates, sulforaphanes, terpenoids, alkaloids, flavonoids, phytosterols, phytoestrogens, and indoles^2^. *P. pudica*, a member of the Apocynaceae family and commonly known as Nag Champa and White frangipani, is not only prized for its beauty but also for its potential medicinal properties. Originating from tropical regions^3,4^, this plant has been associated with anti-allergic, laxative, carminative, cytotoxic, anti-microbial, anti-inflammatory, and various other medicinal properties. In northeastern Brazil, the plant is utilized in traditional medicine for its pain-relieving properties, although scientific data on its pharmacological effects are sparse. A recent study found that *P. pudica* latex demonstrated both anti-inflammatory and pain-relieving effects^5^. Understanding the phytochemical composition of plants is significant because compounds such as phenolics and flavonoids exhibit antioxidant properties, potentially preventing diseases like cancer and heart disease^6^. Research on latex proteins from *Plumeria pudica* has shown that they can impact key aspects of the inflammatory response, including neutrophil migration and the production of cytokines and inflammatory mediators^7^. In the current investigation, a comprehensive evaluation of this particular plant was undertaken to assess the presence of phytochemicals, utilizing both qualitative and quantitative parameters including LCMS-QTOF analysis. The study encompassed the total content estimation of five distinct phytochemicals, along with a conclusive examination of the plant’s antioxidant potential. Two antioxidant tests, namely the reducing power assay and the DPPH radical scavenging assay, were conducted to accomplish this. Furthermore, the antimicrobial properties of the plant were subject to evaluation. This encompassed an antibacterial assay against four bacterial species utilizing agar well diffusion assay, with the concomitant calculation of minimum inhibitory concentration (MIC). The collective results affirm the prospective applicability and value of this plant, endorsing its considerable potential for further research endeavours.

## Methodology

### Collection of plant material

In April 2024, the fresh leaves of *P. pudica* were collected from various sites in the Gandhinagar district of Gujarat State, located at 23°14’ N latitude and 72°38’ longitude. The plant is commonly grown for its ornamental appearance in domestic households and is easily available in local nurseries, so there’s no need to obtain a license to collect it. Dr. B.A. Jadeja identified the plant, and the voucher specimens (KS15A and KS15B) of the collected leaves were deposited at the Department of Botany, M.D. Science College, Porbandar, Gujarat, India. The leaves were thoroughly cleaned with distilled water, air-dried for 6 days, and then carefully ground into a coarse powder, which was prepared for storage in glass bottles.

### Plant extraction process

Plant extraction was achieved through hot extraction using a Soxhlet apparatus. The powdered leaves were placed in a muslin thimble and within a glass chamber. The solvent was added at a ratio of 1:10 g/mL, and the extraction was performed at various temperatures: 82.3 °C for IPA, 100 °C for aqueous, 60 °C for petroleum ether, and 66 °C for the hydroalcoholic extract (with a volume ratio of 6:4 DW to Methanol). The extracted supernatant was filtered through Whatman filter paper, and dried in the air, and the dry weight of the crude extract was determined, providing information on the yield of the extract^8^.

$${\text{Yield}}\;{\text{of}}\;{\text{plant}}\;{\text{extract}} = \frac{{Weight\;of\;crude\;extract\;obtained\left( {{\text{gram}}} \right)}}{{total\;weight\;of\;plant\;powder\left( {{\text{gram}}} \right)}} \times {1}00$$

### Bioassays

#### Preliminary phytochemical analysis

A 2 mg/mL stock solution was prepared for each plant extract, which was then used to test for the presence of various bioactive compounds, including alkaloids, carbohydrates, terpenoids, flavonoids, phenols, tannins, quinones, saponins, amino acids, and proteins. The phytochemical screening was conducted following the protocols outlined by^9,10^.

#### Quantitative phytochemical analysis

To analyse the phytochemicals, we prepared standard concentration and sample concentration series in triplicates. This involved preparing both the standards and extracts in triplicates and taking the mean value of the absorbance from these triplicates for the results.

#### Total content estimation of flavonoids

The aluminum chloride assay was used to determine the total flavonoid content in the extracts. A stock solution of quercetin was prepared at a concentration of 100 µg/mL with methanol. Subsequently, various concentrations of quercetin (20, 40, 60, 80, and 100 µg/mL) were prepared in a methanolic solution. Methanol was added to make the volume up to 1 ml, and 4 mL of distilled water was added to each test tube. After 5 min, 0.3 mL of 5% NaNO_2_ and 0.3 ml of 10% AlCl_3_ were added. Subsequently, 2 mL of 1 M NaOH was added to the mixture after 6 min. The volume of the mixture was adjusted to 10 mL by promptly adding 4.4 mL of distilled water^11^. The absorbance was taken at 510 nm in the spectrophotometer for the standard series. Various concentrations of the extracts (20, 40, 60, 80, and 100 µg/mL) were prepared according to the procedure used for standard quercetin. The absorbance for each concentration of the extracts was recorded using the same method. The total flavonoid content was expressed as quercetin equivalents using a linear equation based on a standard calibration curve. The regression line Y = mx + B from the calibration curve was used to determine the flavonoid concentration of plant extracts, where Y represents the absorbance (510 nm), X represents the concentration (unknown), and m represents the gradient. With the application of the formula x = (y-b)/m we will get the value of x. The total flavonoid content of the extracts was expressed as milligram quercetin equivalents (QE) per gram of sample. The formula used to calculate the total flavonoid content in all the samples was: C = $x\frac{v}{m}$, where C represents the total flavonoid content (mg QE/g of plant extract), x is the concentration, V is the volume of the extract (µl), and m m denotes the concentration of the crude extract (mg/ml). The total flavonoid content of each sample was determined using this formula.

#### Total content estimation of carbohydrates

Place 100 mg of glucose into a test tube. Add 5 mL of 2.5N HCl and heat the mixture in a water bath for 3 h to hydrolyse. Allow the mixture to cool to room temperature. Then, add solid sodium carbonate (Na_2_CO_3_) gradually until effervescence stops, indicating complete neutralization. Filter the solution and dilute it to a final volume of 100 ml. Pipette 0.02, 0.04, 0.06, 0.08, and 0.1 mg/mL of working standard (Glucose) into separate test tubes. Pipette 0.2 ml of the sample solution into another tube and make up the volume to 1 mL with water. Prepare a blank by using all reagents except the sample. Add 1 mL of phenol to each tube, followed by 5 mL of 96% H_2_SO_4_. Shake the tubes well and then incubate them in a water bath at 25–30 °C for 20 min. In the hot acidic medium, glucose is dehydrated to hydroxymethyl furfural, which reacts with phenol to produce a green-colored compound. Measure the color intensity at 490 nm. The whole process is repeated with the leaf extracts at the same concentrations that have been utilized for the standard. Total carbohydrate content was quantified in terms of GLU equivalents using a linear equation derived from a standard calibration curve^9^. The regression line Y = mx + B from the calibration curve was utilized to ascertain the carbohydrate concentration of the plant extracts, wherein Y denotes Absorbance (490 nm), X denotes Concentration (unknown), and m denotes gradient. With the application of the formula x = (y-b)/m we will get the value of x. Now further calculations of the total protein content in all the samples were conducted using the formula C = (x/V) * m, where C denotes the total carbohydrate content (mg GLUE/g of plant extract), x represents the concentration, V indicates the volume of the extract (µL), and m denotes the concentration of the crude extract (mg/mL)^12^.

#### Total content estimation of proteins

The total protein content of *P. pudica* leaves was determined using the methodology outlined by^13^. The development of the blue color is attributed to the reduction of the phosphomolybdic–phosphotungstic components in the Folin reagent, facilitated by the presence of tryptophan and tyrosine amino acids within the protein structure. Additionally, the color generated through the biuret reaction of the alkaline cupric tartrate with protein was quantified utilizing Lowry’s method.

#### Reagents

- 0.1N NaOH: A solution was prepared by dissolving 0.4 g of NaOH in distilled water (100 mL).
- 15% TCA: A solution prepared by dissolving 15 g of trichloroacetic acid in 100 mL of water.
  - Solution A: Contains 2.0% Na_2_CO_3_ in 0.1N NaOH.
  - Solution B: 0.5% CuSO_4_.5H_2_O in 1% sodium potassium tartrate.
  - Solution C: Prepared by mixing solution A and solution B in a 50:1 ratio at the time of use.
- Solution D: Created by combining one part of the Folin phenol reagent with distilled water at the time of use.
- BSA Solution: A solution containing 0.1 g of bovine serum albumin in 1 Liter of distilled water. Protein estimation was conducted using a standard curve generated with BSA concentrations ranging from 200 µg/mL to 1000 µg/mL. The protein concentration was quantified in terms of quercetin equivalents using a linear equation derived from a standard calibration curve. The regression line Y = mx + B from the calibration curve was utilized to ascertain the protein concentration of the plant extracts, wherein Y denotes Absorbance, X denotes Concentration (unknown), and m denotes gradient.

#### Preparation of sample for total protein content

In the process of preparing plant extracts, five grams of fresh and young leaves were pulverized in 5 mL of 0.1 N NaOH, followed by centrifugation at 3000 rpm for 5 min to isolate the supernatant. The combined supernatants were obtained after subjecting the supernatant to a second round of centrifugation to achieve a final volume of 10 mL. Subsequently, 1 mL of 15% TCA was introduced to 2 mL of the supernatant, which was then incubated for 24 h at 4 °C. Proteins in the supernatant were then precipitated and separated by centrifugation at 5000 rpm for 20 min. After discarding the supernatant, the precipitate was dissolved in 5 mL of 0.1 N NaOH for subsequent protein estimation. The resulting protein extract was thoroughly combined with 5 mL of solution C in a test tube and left for 10 min at room temperature. Following this, 0.5 mL of solution D was added and thoroughly mixed. After the lapse of 30 min, the absorbance was measured at 750 nm against a blank using distilled water to replace the extract. The protein concentration was quantified in terms of BSA equivalents using a linear equation derived from a standard calibration curve. The regression line Y = mx + B from the calibration curve was utilized to ascertain the protein concentration of the plant extracts, wherein Y denotes Absorbance (750 nm), X denotes Concentration (unknown), and m denotes gradient. With the application of the formula x = (y-b)/m we will get the value of x. Now further calculations of the total protein content in all the samples were conducted using the formula C = (x/V) * m, where C denotes the total protein content (mg BSAE/g of plant extract), x represents the concentration, V indicates the volume of the extract (µl), and m denotes the concentration of the crude extract (mg/mL).

#### Total content estimation of phenols

The Total Phenolic content in different leaf extracts of *P. pudica* was evaluated using the modified Folin–Ciocalteau method, as described in a previous study by^14^. A gallic acid standard solution was prepared at a concentration of 100 µg/mL. Additionally, gallic acid solutions in methanol at various concentrations (20, 40, 60, 80, and 100 µg/mL) were prepared. For each concentration, 5 mL of 10% Folin–Ciocalteau reagent and 4 ml of 7% Na_2_CO_3_ were added to 5 ml of the respective gallic acid solution, resulting in a final volume of 10 mL. The solution was vigorously agitated and subsequently incubated for 30 min at 40 °C in a water bath. The absorbance was then quantified at 760 nm relative to a blank sample. Polyphenol concentrations in the plant extracts were determined utilizing the regression line Y = mx + B from the calibration curve, where Y = Absorbance (760 nm), X = Concentration (unknown), and m represents the slope of the calibration curve^15^. Preparation of Samples for total Phenolic content. Various concentrations of the extracts were prepared, ranging from 20 µg/mL to 100 µg/mL. The method used for the gallic acid standard was followed, and the absorbance for each concentration of the extracts was recorded. The total phenolic content of the extracts was determined in milligrams of gallic acid equivalents (GAE) per gram of sample. The total phenolic content in all the samples was calculated using the formula: C = $x\frac{v}{m}$, where C represents the total phenolic content (mg GAE/g of plant extract), x is the concentration, V is the volume of the extract in microliters, and m denotes the concentration of the crude extract (mg/mL).

#### Total content estimation of saponins

The total saponin content (TSC) of the leaves of *Plumeria pudica* Jacq. was assessed using the method outlined by^16^. In this procedure, 250 μL of leaf extracts were combined with 250 μL of 8% (w/v) vanillin and 2.5 mL of 72% (v/v) sulfuric acid. The resulting mixture was incubated at 60 °C for 10 min, then cooled in an ice water bath for another 10 min, and its absorbance was measured at 560 nm. A solution without the extract served as the blank. TSC was reported as diosgenin equivalents (mg DIE/g dry weight of plant extract). The standard series’ absorbance was measured at 544 nm using a spectrophotometer. Various concentrations of the extracts (20, 40, 60, 80, and 100 µg/mL) were prepared following the same procedure used for standard diosgenin. The absorbance for each extract concentration was recorded using an identical method. To quantify the total saponin content as diosgenin equivalents, a linear equation based on a standard calibration curve (Y = mx + B) was utilized. This calibration curve was crucial for determining the saponin concentration of the plant extracts. The formula x = (y-b)/m was employed to calculate the saponin concentration. Additionally, the total saponin content of the extracts was expressed as milligram diosgenin equivalents (DIE) per gram of the sample. The total saponin content in all samples was calculated using the formula C = x/v over m, where C represents the total saponin content (mg DIE/g of extract), x is the concentration, v is the volume of the extract (µL), and m denotes the concentration of the crude extract (mg/mL). This method facilitated the determination of the total saponin content of each sample.

### LC–MS QTOF analysis

The components of *P. pudica* Jacq. leaves were assessed using light chromatography coupled with quadrupole time-of-flight mass spectrometry instrumentation (Agilent 6545 XT Advance bio-LC/QTOF). The liquid chromatography analysis was conducted on the Agilent 1290 infinity 2 LC system, a component of the 6545 XT system. The analytical column employed was the Agilent Advance BIO Peptide Mapping, with dimensions of 2.1 × 150 mm and a particle size of 2.7 µm (p/n 653750-902). The temperature of the column and autosampler was adjusted to 60 °C and 4 °C, respectively. Throughout the liquid chromatography process, 0.1% formic acid in water and 0.1% formic acid in 90% acetonitrile were used as solvents. For mass spectrometry, the gas temperature was maintained at 325 °C. The leaf extracts were prepared at a concentration of 2 mg/mL. These extracts underwent two rounds of dilution for analysis. Initially, the leaf extracts were diluted by combining 100 µl of extracts with 900 µl of methanol. The second dilution involved taking 10 µL of the previous sample and adding 990 µL of methanol. Following this, the sample was centrifuged at 4000 rpm for 10 min. The supernatant from the top of the centrifuge tubes was used for the analysis. Compound identification was performed using the NIST library and Agilent Mass Hunter Bio Confirm B 0.9.

### Antioxidant activities

Owing to the complex nature of phytochemicals, the evaluation of antioxidant activity requires at least two test systems to establish authenticity.

### Reducing power assay

An elevation in absorbance values may indicate the antioxidant capacity of the antioxidants or their respective extracts. Chemical compounds possessing antioxidant properties undergo a reaction with potassium ferricyanide (K_3_[Fe (CN)_6_]), resulting in the formation of potassium ferrocyanide (K_4_[Fe (CN)_6_]). The resultant compound subsequently reacts with ferric trichloride, yielding ferric ferrocyanide, characterized by a blue-colored complex exhibiting a peak absorbance at 700 nm^17^. The preparation of the sample solution involved the utilization of plant extracts at concentrations of 200, 400, 600, 800, and 1000 µg/mL, subsequently combined with 1 mL of distilled water. This mixture was then supplemented with 2.5 mL of 0.2 M pH 6.6 phosphate buffer and 2.5 mL of 1% potassium ferricyanide [K_3_Fe (CN)_6_]. Following this, the mixtures were incubated at 50 °C for 20 min. After this incubation period, 2.5 mL aliquots of trichloroacetic acid (10%) were added to each mixture, and then centrifuged for 10 min at 1036 × g. The upper layer of these solutions (2.5 mL) was then separately mixed with 2.5 mL of distilled water and 0.5 mL of 0.1% FeCl_3_, and the absorbance was measured at 700 nm using a spectrophotometer. Methanol was employed as a control. Ascorbic acid served as the positive control.

### DPPH assay

The antioxidant activity of the extracts was measured using the DPPH free radical scavenging assay, with slight modifications to the method described previously^18^. DPPH, in its oxidized state, appears as a deep violet color in methanol. When an antioxidant compound is introduced, it donates an electron to DPPH, causing its reduction and a color change from violet to blue to yellow. DPPH solutions have an absorbance of 517 nm, and the scavenging of DPPH free radicals determines the free radical scavenging capacity and antioxidant potential of plant samples. Preparation of DPPH solution (0.1 M). DPPH solution was prepared by dissolving 0.39 mg of DPPH reagent in methanol in a volumetric flask, and the final volume was approximately 100 mL. The purple-colored DPPH solution was stored in a freezer at -15 °C for further use.

### Preparation of extract solutions

Stock solutions of 2 mg/mL extracts were prepared by dissolving each extract in methanol, followed by dilution to 20, 40, 60, 80, and 100 µg/mL. To assess antioxidant potential, these sample solutions were mixed with 1 mL of DPPH solution and incubated in darkness at room temperature for 30 min. A control solution of 1 mL methanol and 1 mL DPPH was also prepared. After incubation, absorbance was measured at 517 nm using a spectrophotometer, with ascorbic acid as the standard. The IC_50_ values of the extracts were determined from the concentration versus percentage inhibition graph, and the DPPH free radical inhibition percentage was calculated using a specific formula.

$${\text{Inhibition}}\;\left( \% \right) = \frac{{A_{control} - A_{test} }}{{A_{control} }} \times 100$$

where A_control_ is the absorbance of the control, and the A_test_ is the absorbance of the reaction mixture samples (in the presence of the sample). Each test was conducted in triplicate (n = 3), and the average values were calculated.

### Ic_50_ value

In the assessment of the DPPH method,^19^ employed the inhibition concentration (IC_50_) parameter. A plot of sample discoloration against sample concentration was generated to ascertain the IC_50_ value. This value denotes the quantity of sample necessary to achieve a 50% reduction in the absorbance of DPPH.

### Antimicrobial assay

Tested microorganisms and preparation of cultures: The antimicrobial potential of *P. pudica* leaf extracts were tested using the agar well diffusion method as described by^20^. The antimicrobial activity was assessed against three microorganisms: *Kocuria rhizophila* ATCC 9341 (gram-positive), *Pseudomonas aeruginosa* ATCC 27853, *Klebsiella pneumoniae* ATCC 13883, and *E. coli* ATCC 25922 (gram-negative), obtained from Pure Microbes, Pune. Bacterial cultures were used to prepare inoculates with a cell density of 10^6 cells/ml using the direct colony suspension method. Sterilized Petri plates with Mueller–Hinton agar were prepared. Leaf extracts were obtained using four solvents—Isopropyl alcohol, aqueous, petroleum ether, and hydro alcohol—to extract polar, medium polar, and nonpolar bioactive components. The crude extracts were dissolved in DMSO. A sterile cork borer was used to create 6 mm diameter wells in each agar plate, filled with 100 µL of plant extract solution at varying concentrations. A 5% DMSO solution served as the negative control. Plates were incubated at 37 °C for 18 h^21^. After incubation, the diameter of the zone of inhibition around each well was measured using microbial calipers.

### Calculation of zone of inhibition and MIC

The agar plate dilution method was used to determine the minimum inhibitory concentration (MIC) of plant extracts. Extracts were tested at concentrations of 10, 15, 20, and 25 mg/mL. Bacterial cultures, grown overnight in broth to a concentration of 10^8^ CFU/mL, were placed onto the agar plates and incubated at 37 °C for 24–48 h. The lowest concentration of each extract that inhibited bacterial growth on Mueller–Hinton agar was recorded as the MIC, expressed in mg/mL^22,23^.

## Results

### Morphological natalities

**Fig. 1:**
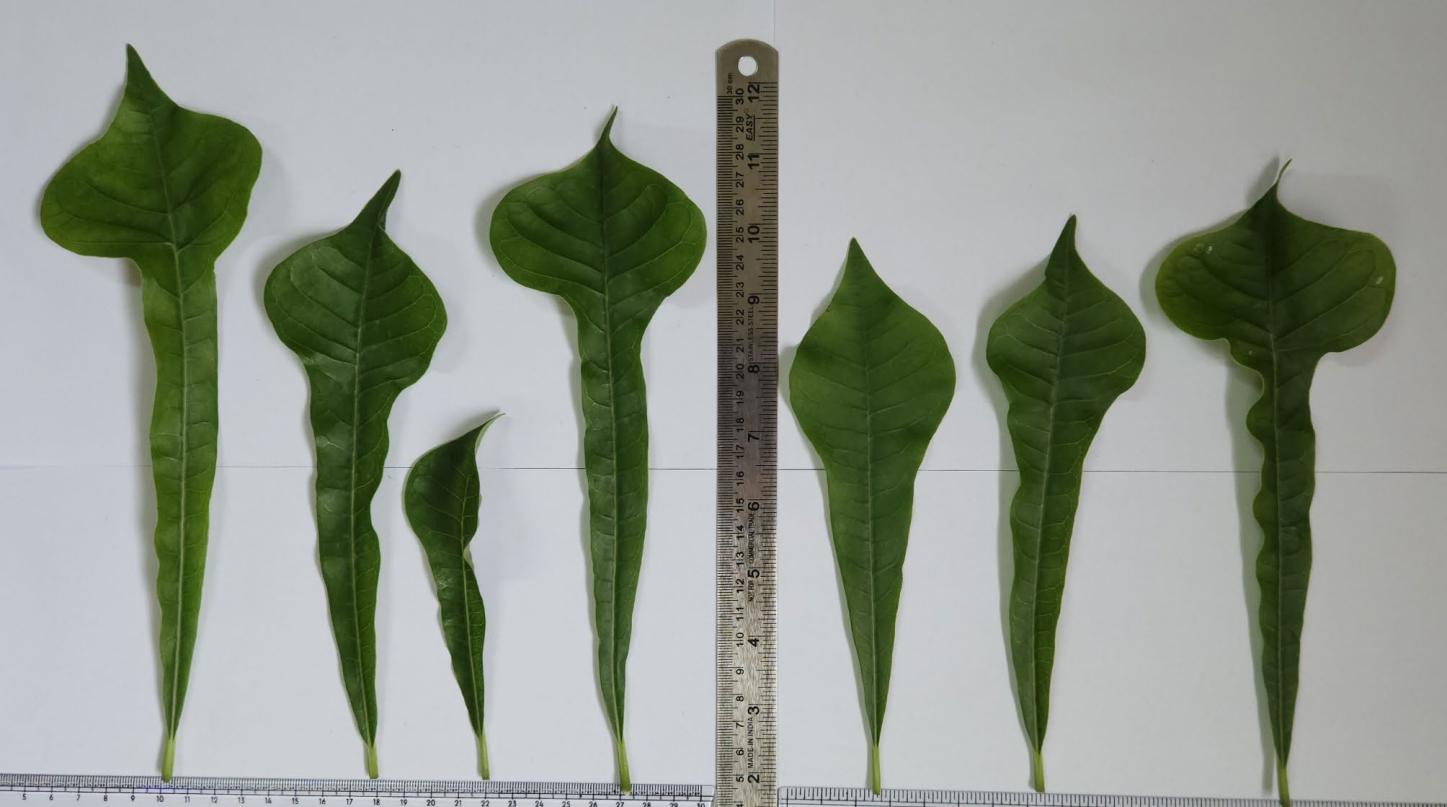
Differences among leaves of *P. pudica* with different-shaped lobes and with different thicknesses of mid-zone.

**Fig. 2:**
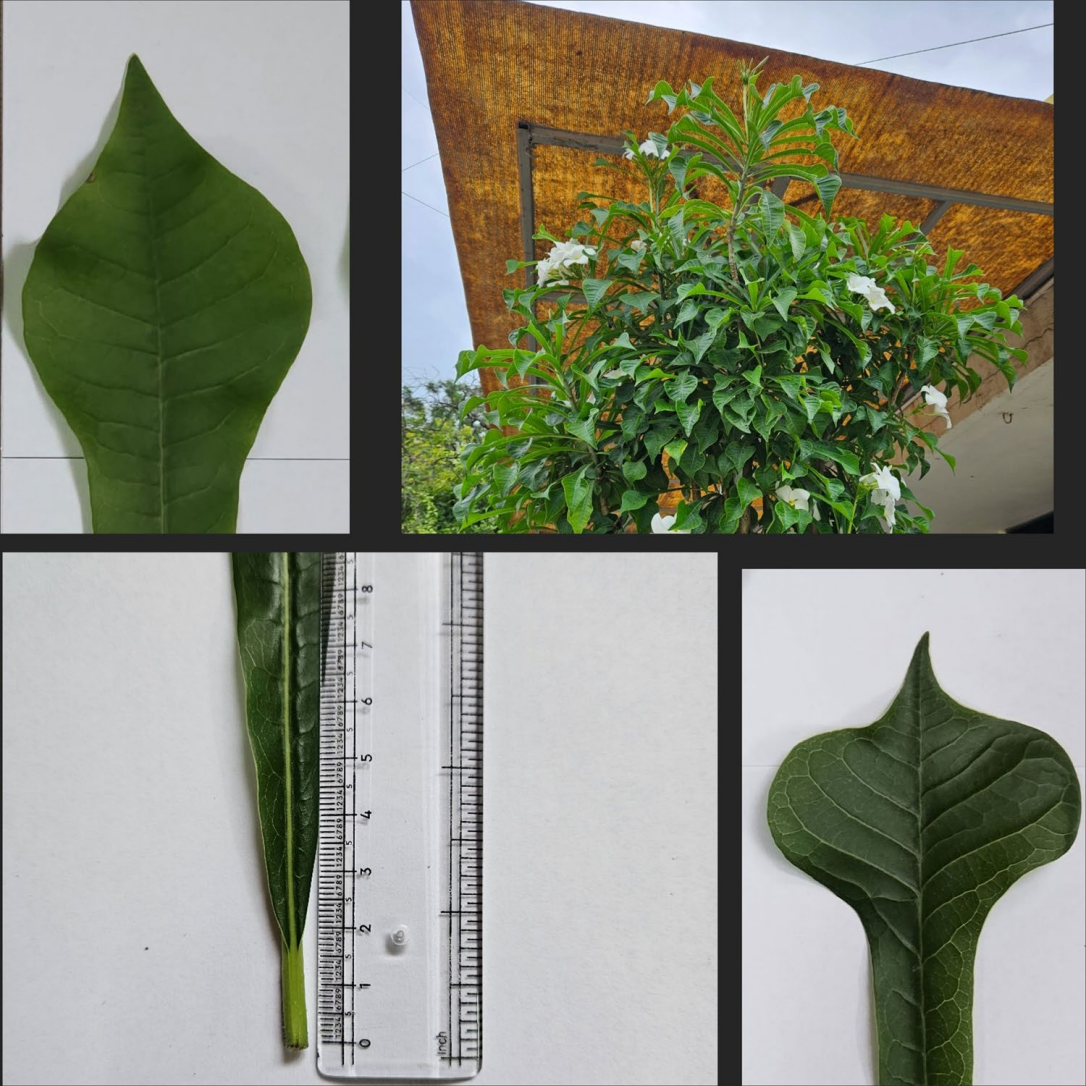
Morphological features of leaves of *P. pudica.*

**Fig. 3:**
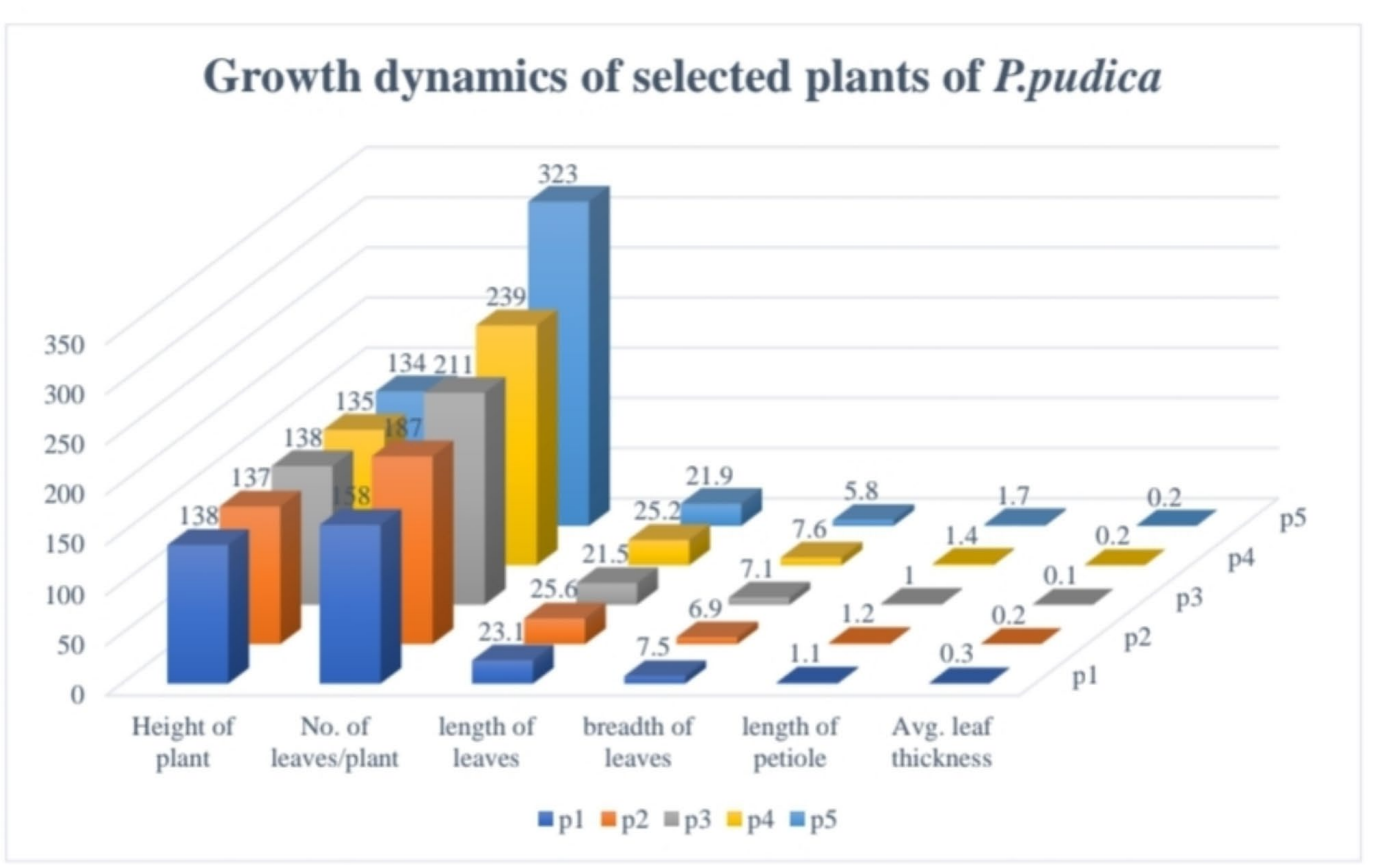
The growth dynamics of the five selected plants of *P. pudica* from various locations across Gujarat.

Five different abscissions of *P. pudica* were collected from five different locations across Gujarat. The first plant was collected from Indroda Park, Gandhinagar City (P1), second from Mahudi town in Mansa taluka (P2), third from Vijapur City (P3), fourth from Dharampur district, Valsad (P4), and fifth from Ahmedabad city (P5). All the selected plants were healthy and were of the same average height (135 ± 3 cm). These five plants were observed for the macro differences in terms of morphological structures of the leaves (Figs. 1, 2). The plant is propagated and multiplied using cuttings, rather than seeds. Although the plant can reach a height of 14 to 18 feet, it cannot be considered a tree because plants that tall have a very small canopy coverage, typically only 1/3 of their height. Furthermore, our research has shown that the number of leaves on the plant varies regardless of its height. As shown in Fig. 3 we observed the five plants from different locations with the same average height of 135 ± 3 cm, plant P1 showed the presence of 158 leaves but plant P5 showed the presence of 323 leaves. Also, there was a variance of 4 cm in the length of leaves as the plant P3 leaf showed a length of 21.5 cm and the leaf of P2 showed length of 25.6 cm (All the leaves that are taken into consideration are selected from the middle part of the canopy where the leaves attained maximum growth). The breadth of leaves showed a variance of almost two centimeters as the leaf of plant P5 has a breadth of 5.8 cm whereas the leaf of plant P1 has a breadth of 7.5 cm. The variations in the leaves among the five selected plants could be attributed to agroclimatic conditions, nutrition, and geology of the soil. This particular plant is an exquisite ornamental species that is predominantly grown in domestic settings, hotels, and various other establishments. It does not naturally occur in the wild. These observations are incredibly beneficial due to the remarkable similarities in appearance, including the leaves and flowers, shared among other members of the Plumeria genus (Table 1). Consequently, this research provides valuable assistance in easily identifying this distinct plant.

**Table 1:** Morphological features of leaves of *P. pudica*.

Leaf color	Dark green to parrot green
Leaf growth on the stem	In an acropetal manner
Texture of leaf	Smooth
Shape of leaf	Distinct fiddle shaped with two lobes in the middle, some spoon-shaped
Leaf base shape	Cuneate
Leaf apex shape	Caudate
Venation	Pinnate with reticulate

### Yield of extracts

The yield value was used to quantify the crude dry powder of the plant part employed. As depicted in Table 2, the highest yield of 87.83% was achieved for the aqueous extract, followed by a 52.89% yield for the hydroalcoholic extract, 10.38%, and 6.38% for the IPA and petroleum extracts, respectively.

**Table 2:** Yield of extracts for the leaves of *P. pudica*.

Extract	% Yield	Colour of crude	pH
IPA extract	10.38	Parrot green	6.5
Hydroalcoholic extract	52.89	Dark green	7.5
Petroleum ether extract	6.38	Brownish green	6.8
Aqueous extract	87.83	Blackish green	6.9

### Qualitative phytochemical analysis

The extraction efficiency of four solvents—IPA, aqueous, petroleum ether, and hydroalcoholic—was assessed based on their polarity (Table 3). All leaf extracts contained carbohydrates, terpenoids, flavonoids, phenols, and proteins. The petroleum ether extract, being non-polar, showed the absence of alkaloids, tannins, quinones, and saponins. Alkaloids were present only in the IPA extract, as all three other extracts (aqueous, petroleum ether, and hydroalcoholic) lacked alkaloids.

**Table 3:** Qualitative Phytochemical Analysis for the Leaves of *P. pudica*.

Phytochemicals	Tests	PP1	PP2	PP3	PP4
Carbohydrates	Benedict’s test	+	+	+	+
Alkaloids	Mayer’s test	+	-	-	-
Terpenoids	Slowaski test	+	+	+	+
Flavonoids	Lead acetate test	+	+	+	+
Phenols	Ferric chloride test	+	+	+	+
Tannins	Folin Ciocalteau test	+	+	-	+
Quinones	Hydrochloric acid test	+	+	-	+
Amino acids and proteins	Biuret test	+	+	+	+
Saponins	Foam test	+	+	-	+

PP1: IPA extract of leaves.

PP2: Aqueous extract of leaves.

PP3: Petroleum ether extract of leaves.

PP4: Hydroalcoholic extract of leaves.

### Quantitative phytochemical analysis

As per the values described in Table 4, we get an idea of the total contents of the various phytochemical compounds present in the leaf extracts of the plant. Also, a comparative representation of the total content of the compounds exhibited by various leaf extracts has been given in Fig. 9.

**Table 4:** Total content estimation of various phytochemicals for the leaves of *P. pudica.*

Phytochemical	Standard	Measures in	PP1	PP2	PP3	PP4
Flavonoids	Quercetin (100 µg/mL)	MG QE/GM	402.20	178.5	65.54	79.85
Carbohydrates	Glucose (100 µg/mL)	MG GLUE/GM	336	207.21	153.07	226.6
Protein	BSA (1000 µg/mL)	MG BSAE/GM	164	157.2	122	115.8
Phenols	Gallic acid (100 µg/mL)	MG GAE/GM	101	98	50.75	57
Saponins	Diosgenin (100 µg/mL)	MG DIE/GM	125.3	108.41	-	202

PP1: IPA extract.

PP2: Aqueous extract.

PP3: Petroleum ether extract.

PP4: Hydroalcoholic extract.

### Total flavonoid content

**Fig. 4:**
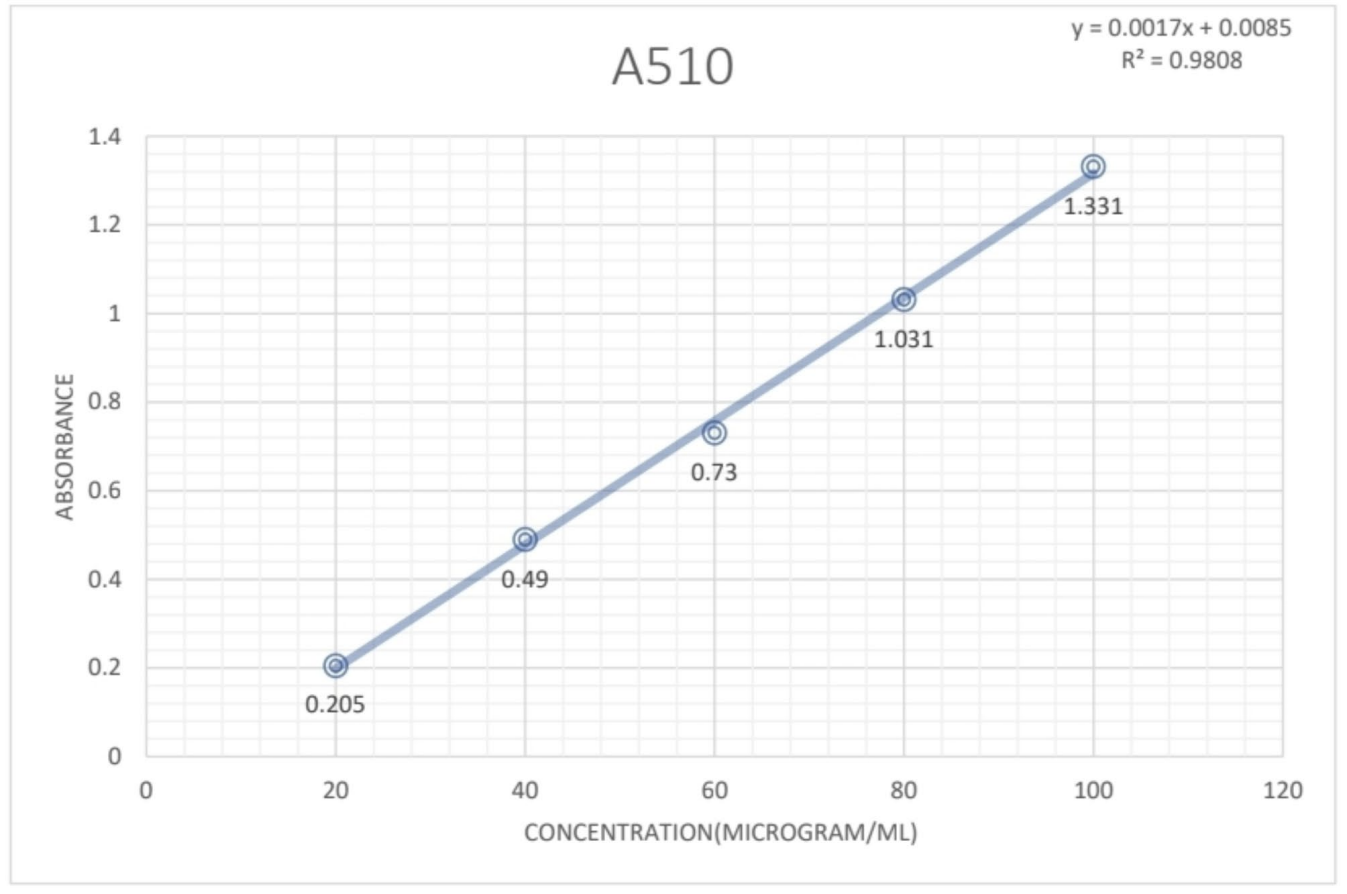
The standard curve for Quercetin for estimation of total Flavonoid content of *P. pudica* leaves.

The total flavonoid content in four extracts of leaf of *P. pudica* was determined by the aluminum chloride method using quercetin as standard. The absorbance values obtained at different concentrations of quercetin were used for the construction of a calibration curve. The total flavonoid content of the extracts was calculated from the regression equation of the calibration curve (Fig. 4) from which the following equations were derived:

$${\text{Y}} = 0.00{17} + 0.00{85}$$

$${\text{R}}^{{2}} = 0.{98}0$$

The highest TFC content was observed in the IPA extract of leaf which was 402.2 mg QE/g of dry-weight of plant extract, which was followed by Aqueous leaf extract with 178.5 mg QE/g dry-weight of plant extract. of TFC and Hydroalcoholic leaf extract with around 79.85 mg QE/g dry-weight of plant extract of TFC. In the petroleum ether extract of the leaf, the least flavonoid content was observed which was 65.54 mg QE/g dry-weight of plant extract.

### Total carbohydrate content

**Fig. 5:**
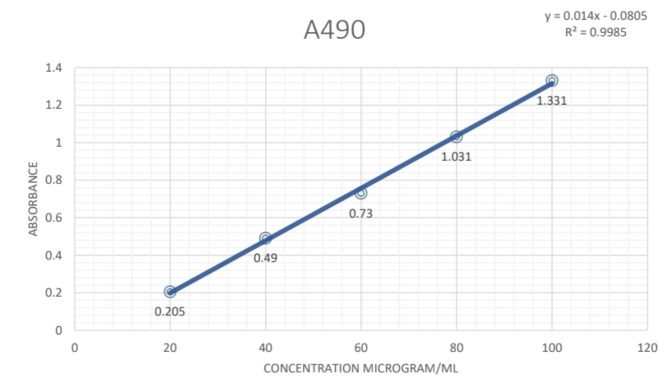
The standard curve for Glucose for estimation of total Carbohydrate content of *P. pudica* leaves.

The total carbohydrate content of the leaves of *Plumeria pudica* Jacq. was determined using glucose as standard. The absorbance values obtained at various concentrations of quercetin were used to create a calibration curve (Fig. 5). The total flavonoid content of the extracts was calculated from the regression equation of the calibration curve. The value of the equation Y = mx + B of the calibration curve is as following:

$${\text{Y}} = 0.0{\text{14x}} - 0.0{8}0{5}$$

$${\text{R}}^{{2}} = 0.{9985}$$

The carbohydrate content of maximum value was observed in the IPA extract of the leaf which was 336 mg GLUE/g dry-weight of plant extract. followed by HYA extract of the leaf with 226.6 mg GLUE/g dry-weight of plant extract, Aqueous extract of the leaf with 207.21 mg GLUE/g dry-weight of plant extract, and the least amount of carbohydrates were present in Petroleum ether leaf extract with 153.07 mg GLUE/g dry-weight of plant of extract.

### Total protein content

**Fig. 6:**
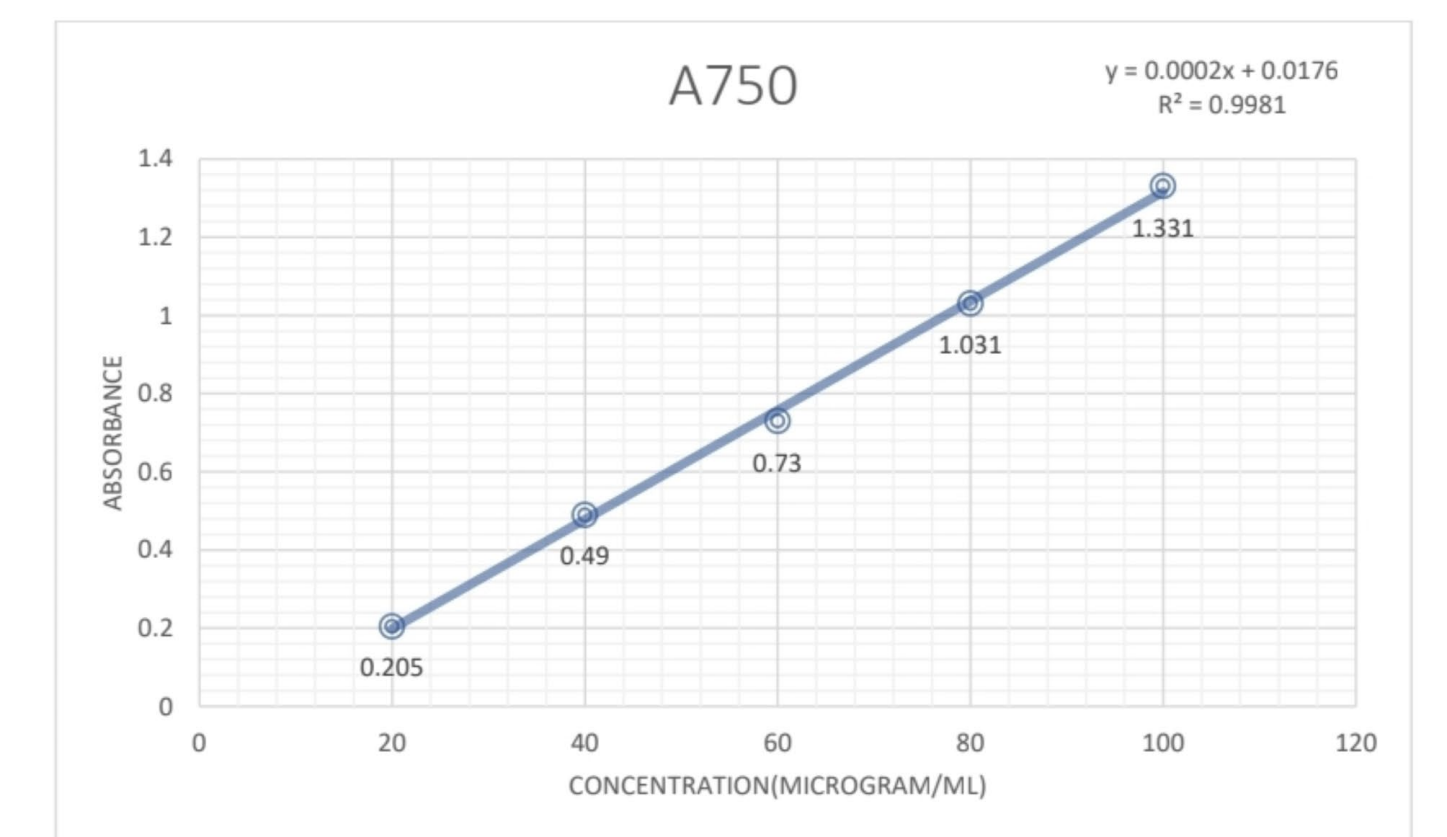
The standard curve for BSA for estimation of total Protein content of *P. pudica* leaves.

Total protein content in four extracts of leaf of *Plumeria pudica* Jacq. were determined by Lawrey’s method using BSA (Bovine Serum Albumin) as standard. The absorbance values measured across various concentrations of BSA were utilized to establish a calibration curve. The total protein content of the extracts was calculated from the regression equation of the calibration curve (Fig. 6) from which the following equations were derived:

$${\text{Y}} = 0.000{2} + 0.0{176}$$

$${\text{R}}^{{2}} :0.{9981}$$

The highest total protein content was observed in the IPA extract of leaf which was 164 mg BSAE/g dry-weight of plant extract of Total protein content which was followed by Aqueous leaf extract with 157.2 mg BSAE/g dry-weight of plant extract of Total protein content and Petroleum ether leaf extract with around 122 mg BSAE/g dry-weight of plant extract of Total protein content. The least amount of Total protein content was observed in the Hydroalcoholic extract which was 115.8 mg BSAE/g dry-weight of plant extract.

### Total phenol content

**Fig. 7:**
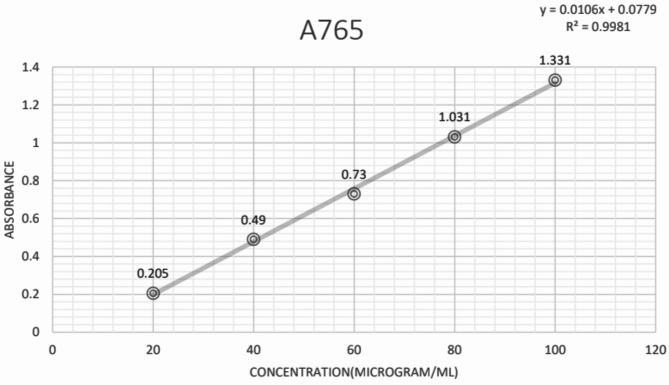
The standard curve for Gallic acid for estimation of total Phenolic content of *P. pudica* leaves.

The content of total phenolics in four leaf extracts of *P. pudica* was assessed using the Folin–Ciocalteau method and gallic acid as the standard. A calibration curve was established by plotting absorbance values from various concentrations of gallic acid. The total phenolic content of the extracts was calculated using the regression equation derived from the calibration curve (Fig. 7), which yielded the following equations:

$${\text{Y}}\, = \,0.0{1}0{6}\, + \,0.0{779}$$

$${\text{R}}^{{2}} \, = \,0.{9981}$$

The highest levels of total phenolic content were found in the IPA extract of leaves, amounting to 101 mg GAE/g dry-weight of plant extract of TPC, followed by the aqueous leaf extract with 98 mg GAE/g dry-weight of plant extract of TPC, and the Hydroalcoholic leaf extract with 57 mg GAE/g dry-weight of plant extract of TPC. Meanwhile, the lowest total phenolic content was observed in the petroleum ether extract of the leaf, amounting to approximately 50.75 mg GAE/g dry-weight of plant extract.

### Total saponin content

**Fig. 8:**
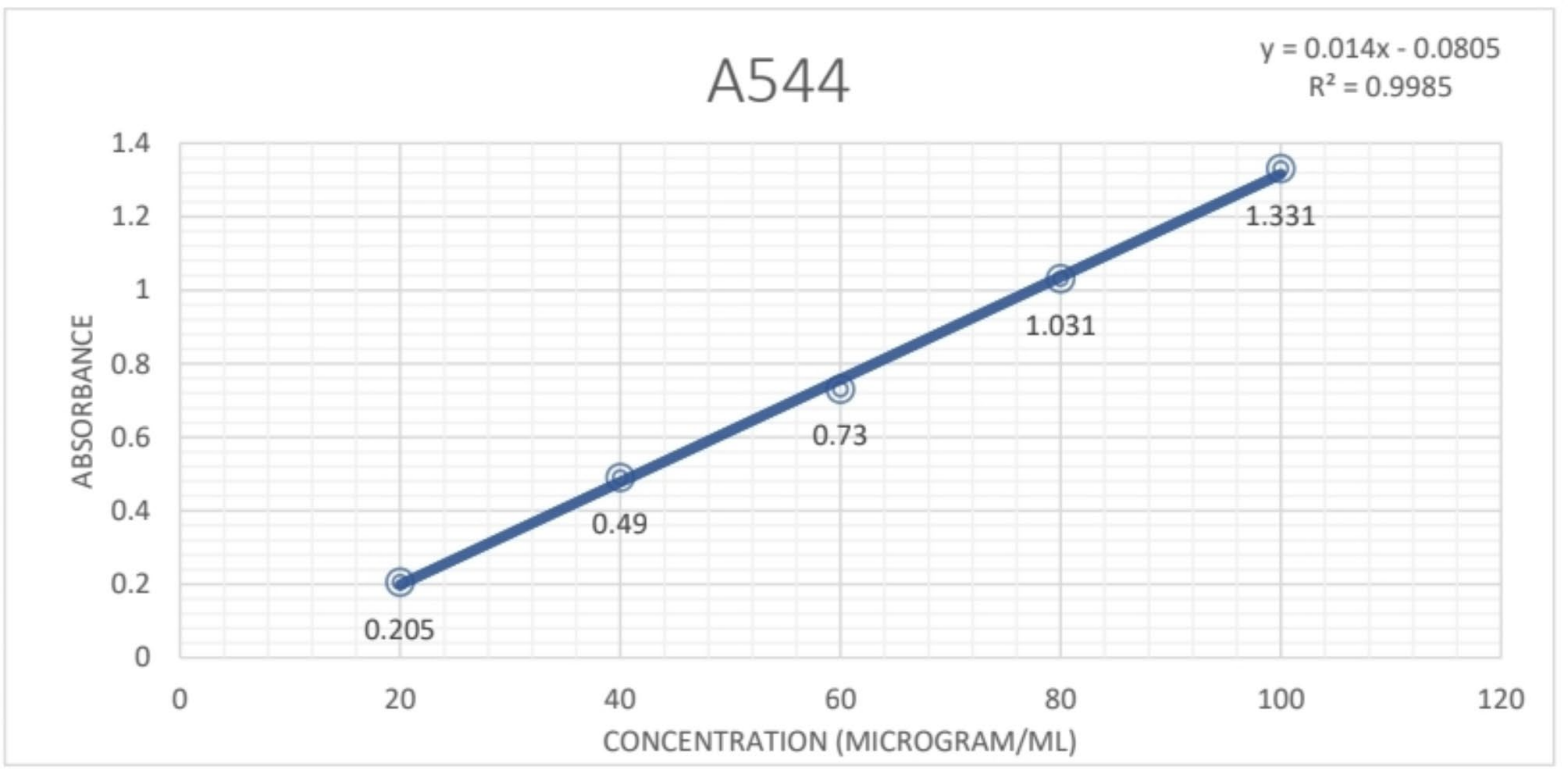
The standard curve for Diosgenin for estimation of total Saponin content of *P. pudica* leaves.

The total saponin content in four extracts of the leaf of *P. pudica* was determined by the Vanillin sulphuric acid method using diosgenin as standard. The absorbance values obtained at various concentrations of diosgenin were utilized to create a calibration curve. The total saponin content of the extracts was calculated from the regression equation of the calibration curve (Fig. 8) from which the following equations were derived:

$${\text{Y}}\, = \,0.00{13}\, + \,0.00{83}$$

$${\text{R}}^{{2}} \, = \,0.{9988}$$

The highest Total saponin content was observed in the hydro alcohol extract of the leaf which was 202 mg DIE/g dry-weight of plant extract. This was followed by IPA leaf extract with 125.3 mg DIE/g dry-weight of plant extract and Aqueous leaf extract with around 108.41 mg DIE/g dry-weight of plant extract. In the petroleum ether extract of the leaf, no saponin content was observed in the preliminary phytochemical analysis so the total saponin content measure was refrained in the petroleum ether extract of the leaf.

### Results of LCMS-QTOF analysis

**Fig. 9:**
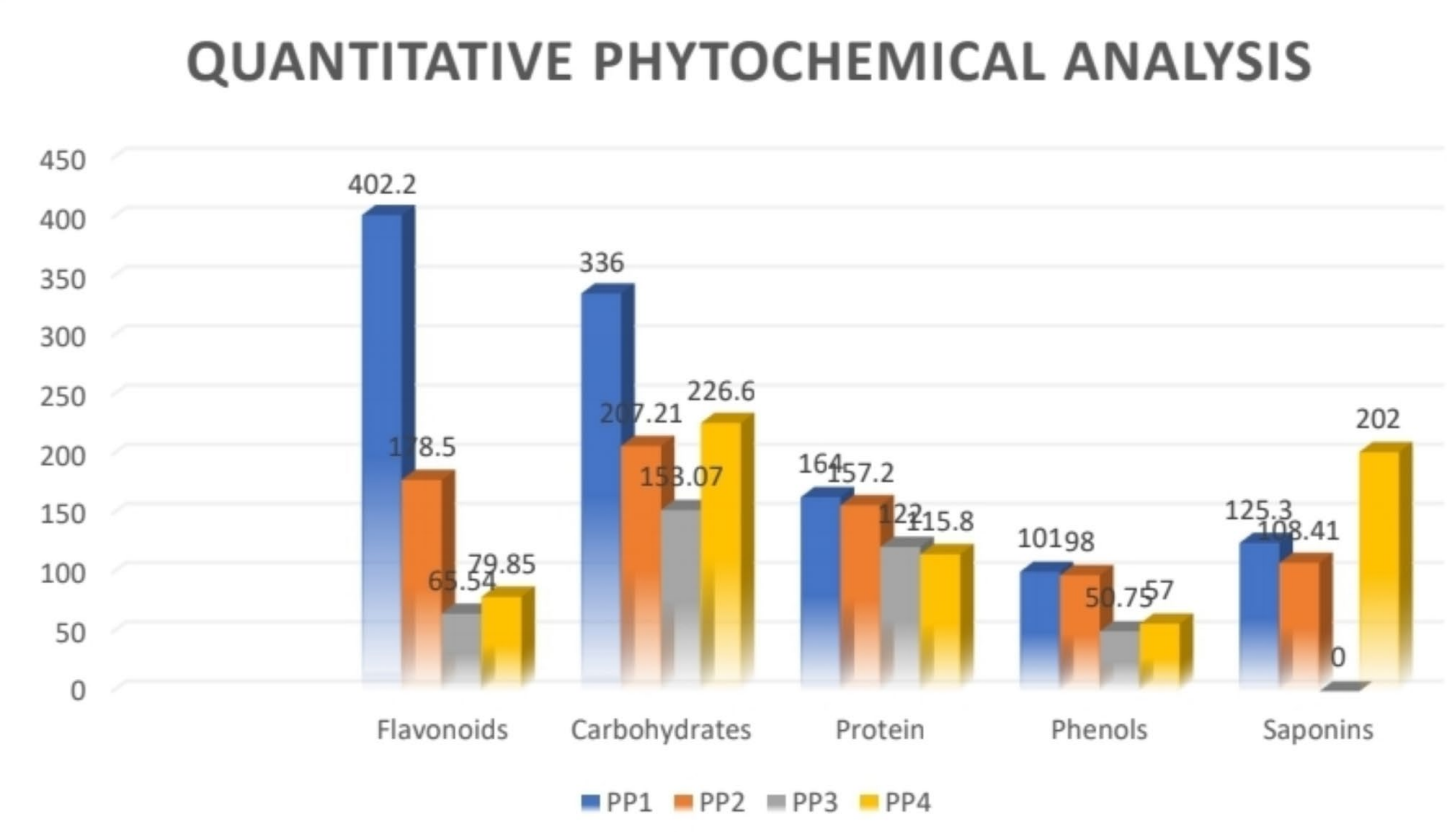
Total contents of various phytochemicals in leaves of *Plumeria pudica* Jacq. (PP1: IPA extract, PP2: Aqueous extract, PP3: Petroleum ether extract, PP4: Hydroalcoholic extract).

The analysis of leaf extracts from *P. pudica* revealed the presence of 24 different compounds in the IPA extract, including important phytochemicals such as Coumarin, Olprinone, Visnagine, and Plumieride (Tables 5, 6, 7 and 8). The aqueous leaf extract consisted of 20 different compounds, including compounds used as drugs such as Synthalin A and Remoxipride. The petroleum ether extract contained around 15 compounds, while the hydroalcoholic extract contained 17 compounds. Many of the detected compounds have pharmaceutical, industrial, and antimicrobial significance. Therefore, the antimicrobial and antioxidant activities of this plant may be attributed to these compounds, highlighting the plant’s importance (Fig. 9).

**Table 5:** The chemical composition of the IPA leaf extract as evaluated by LCMS-QTOF analysis.

Peak	Retention time	Mass/charge ratio	Molecular weight (g/mol)	Structure	IUPAC name of the proposed compound
1	1.551	118.0867	117.15	C5H11NO2	Valine
2	1.85	144.1019	143.18	C7H13NO2	Stachydrine
3	2.448	215.0162	192.12	C6H807	Citric acid
4	5.802	119.0492	118.13	C8H6O	Benzofuran
5	5.9	147.0441	146.14	C9H6O2	Coumarin
6	5.963	165.0546	164.16	C9H8O3	Coumarinic acid
7	6.278	305.0993	304.25	C11H16N2O8	Spaglumic acid
8	7.178	231.0652	230.22	C13H10O4	Visnagine
9	7.406	273.0758	250.25	C14H10N4O	Olprinone
10	7.539	291.0863	290.27	C15H1406	Epicatechin
11	8.014	493.1314	470.4	C21H26O12	Plumieride
12	8.580	147.0441	146.14	C9H6O2	Phenylpropinoic acid
13	9.016	231.0652	230.22	C13H10O4	Visnagidin
14	9.322	274.2741	273.45	C16H35N02	Lauryl diethanolamine
15	9.492	291.865	290.27	C15H14O6	Cianidanol
16	10.325	112.1247	112.21	C8H16	Octylene
17	10.381	168.1873	168.32	C12H24	Cyclododacane
18	10.437	182.2029	182.35	C13H26	1-tridecene
19	10.493	238.2655	238.5	C17H34	Heptadecene
20	14.018	149.0237	148.11	C8H4O3	Phthalic anhydride
21	14.048	338.3420	319.4	C22H23O2	CID 101241023
22	14.053	391.2843	390.6	C24H38O4	Diiethylhexylpthalate
23	14.113	485.2896	484.6	C29H40O6	Stigmatellin Y
24	14.143	530.3475	512.7	C31H44O6	Carindone

**Table 6:** The chemical composition of the Aqueous leaf extract as evaluated by LCMS-QTOF analysis.

Peak	Retention time	Mass/charge ratio	Molecular weight (g/mol)	Structure	IUPAC name of the proposed compound
1	1.27896	104.1061	86.12	C3H8N3	Azanium
2	1.35	130.1577	112.21	C8H16	1,3-dimethyl cyclohexane
3	1.445	194.1155	119.38	C13H29N	Tridecan-2-amine
4	1.49	302.1927	164.35	C9H24S	Ethane
5	6.945	130.1579	112.21	C8H16	Cyclooctane
6	9.545	130.1579	112.21	C8H16	Cyclooctane
7	9.568	200.2354	182.35	C13H26	Cyclotridecane
8	9.604	270.3128	252.5	C18H36	1-octadecene
9	9.618	274.2711	256.39	C12H28N6	Synthalin A
10	11.310	130.1579	112.21	C8H16	1-octene
11	11.345	200.2354	182.35	C13H26	1-tridecene
12	11.381	270.3129	252.5	C18H36	Dodecylcylohexane
13	11.501	352.3362	-	C11H34N12	Not identified
14	11.549	540.5289	-	C35H71O5	Not identified
15	12.168	371.0974	289.31	C15H17N2O4	6-hydroxy-IAA-valine
16	12.598	371.0980	371.27	C16H23BRN2O3	Remoxipride
17	13.154	149.0221	148.10	C6H2N3O2	2-cyanopyrimidine-5-carboxylate
18	13.167	311.3100	-	C11H33N8O	Not identified
19	13.178	411.3438	-	C19H40N9O	Not identified
20	13.183	568.5617	424.8	C30H64	Ethane

**Table 7:** The chemical composition of the Petroleum ether leaf extract as evaluated by LCMS-QTOF analysis.

Peak	Retention time	Mass/charge ratio	Molecular weight (g/mol)	Structure	IUPAC name of the proposed compound
1	1.275	104.1061	86.12	C3H8N3	CID 59774579
2	1.435	194.1155	193.23	C9H13N4O	CID 57447885
3	1.485	302.1927		C19H24S	2-hexyl-4-methyl-1-benzene
4	2.115	116.1434	98.19	C7H14	1-Heptene
5	2.223	149.0220	148.10	C6H2N3O2	2-cyanopyrimidine-5-carboxylate
6	2.489	214.2152	468.16	C11H26I2N4	Piperidium
7	6.941	130.1579	112.21	C8H16	1-Octene
8	9.532	130.1582	112.21	C8H16	Caprylene
9	9.568	200.2354	182.35	C13H26	Cyclotridecane
10	11.389	270.3129	252.5	C18H36	1-octadecene
11	11.532	352.3362	-	C11H34N12	Not identified
12	12.184	540.5291	-	C35h71OS	Not identified
13	12.517	371.0965	371.27	C16H23BRN2O3	Remoxipride
14	13.174	149.0221	136.09	C6H2N3O2	CID74441148
15	13.239	311.3100	-	C11H33N8O	Not identified

**Table 8:** The chemical composition of the Hydroalcoholic leaf extract as evaluated by LCMS-QTOF analysis.

Peak	Retention time	Mass/charge ratio	Molecular weight (g/mol)	Structure	IUPAC name of the proposed compound
1	1.551	118.0867	117.15	C5H11NO2	Valine
2	1.85	144.1019	143.18	C7H13NO2	Stachydrine
3	2.448	215.0162	192.12	C6H807	Citric acid
4	5.802	119.0492	118.13	C8H6O	Benzofuran
5	5.9	147.0441	146.14	C9H6O2	Coumarin
6	5.963	165.0546	164.16	C9H8O3	Coumarinic acid
7	6.278	305.0993	304.25	C11H16N2O8	Spaglumic acid
8	7.178	231.0652	230.22	C13H10O4	Visnagine
9	7.406	273.0758	250.25	C14H10N4O	Olprinone
10	7.539	291.0863	290.27	C15H1406	Epicatechin
11	8.014	493.1314	470.4	C21H26O12	Plumieride
12	8.580	147.0441	146.14	C9H6O2	Phenylpropinoic acid
13	9.016	231.0652	230.22	C13H10O4	Visnagidin
14	9.322	274.2741	273.45	C16H35N02	Lauryl diethanolamine
15	9.492	291.865	290.27	C15H14O6	Cianidanol
16	10.325	112.1247	112.21	C8H16	Octylene
17	10.381	168.1873	168.32	C12H24	Cyclododacane

### In vitro antioxidant activity

#### Reducing power assay

**Fig. 10:**
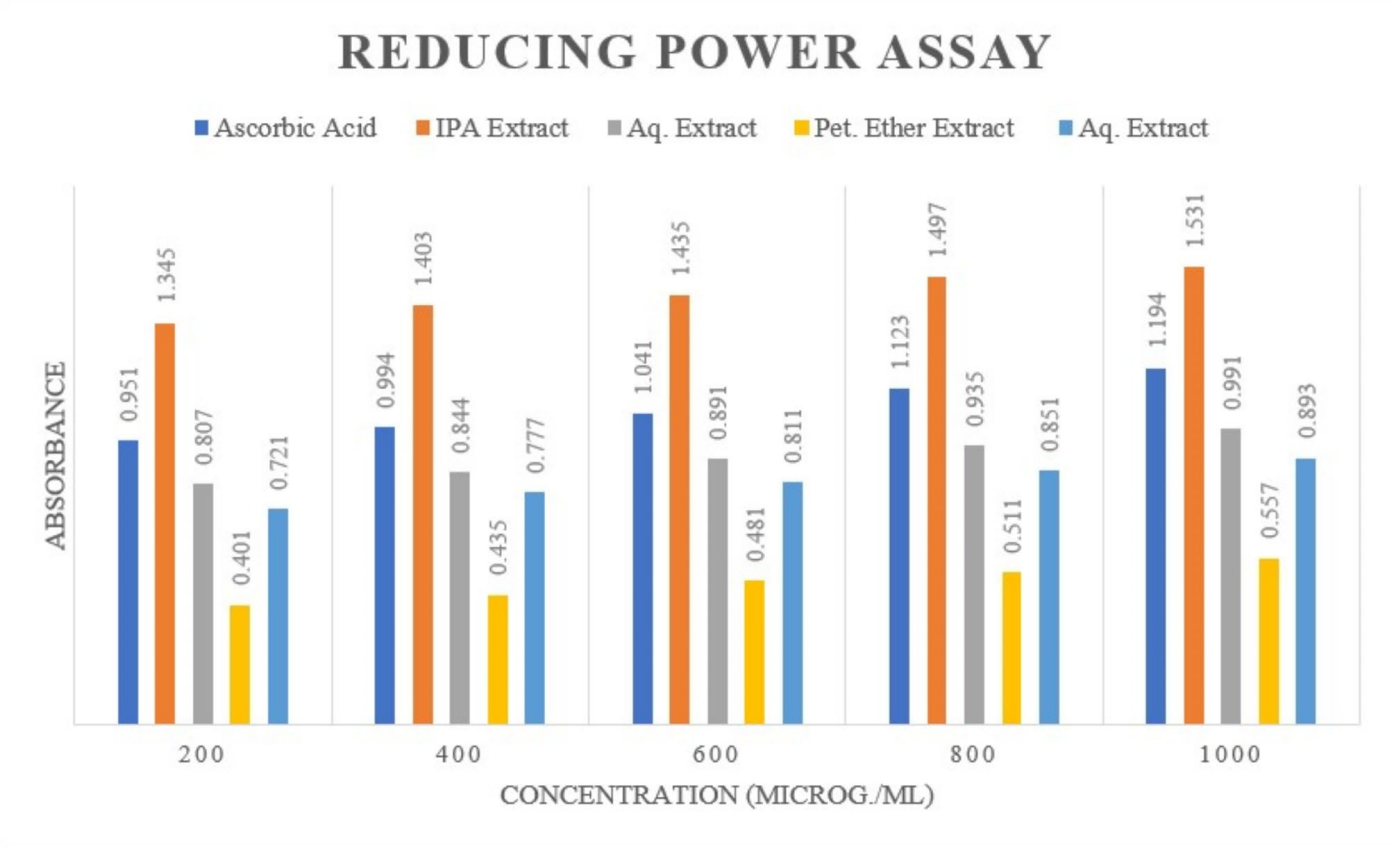
Comparison of absorbance at 700 nm for standard and leaf extracts of *P. pudica* (Concentration in µg/mL) for reducing power assay.

Antioxidant potential is closely linked to reducing power, which serves as a significant indicator of this potential. Compounds with good reducing power act as electron donors and effectively counteract oxidized intermediates in lipid peroxidation processes. During the assay, the color change from dark green to various shades of blue reflected the distinct reducing power of each compound. Reducing agents prompt the conversion of the Fe^3+^/ferricyanide complex to its ferrous form, with absorbance measured at 700 nm indicating the ferrous ion concentration. A higher absorbance in the reaction mixture signified an increased reducing power of the extracts. As shown in Fig. 10, at the highest concentration (1000 µg/mL), ascorbic acid exhibited an absorbance of 1.19, while the IPA extract showed the highest absorbance at 1.53, followed by the Aqueous extract at 0.99, the Hydroalcoholic extract at 0.89, and the lowest absorbance of 0.55 with petroleum ether extract. This suggests the excellent antioxidant properties of the IPA and Aqueous extracts.

#### DPPH assay

**Fig. 11:**
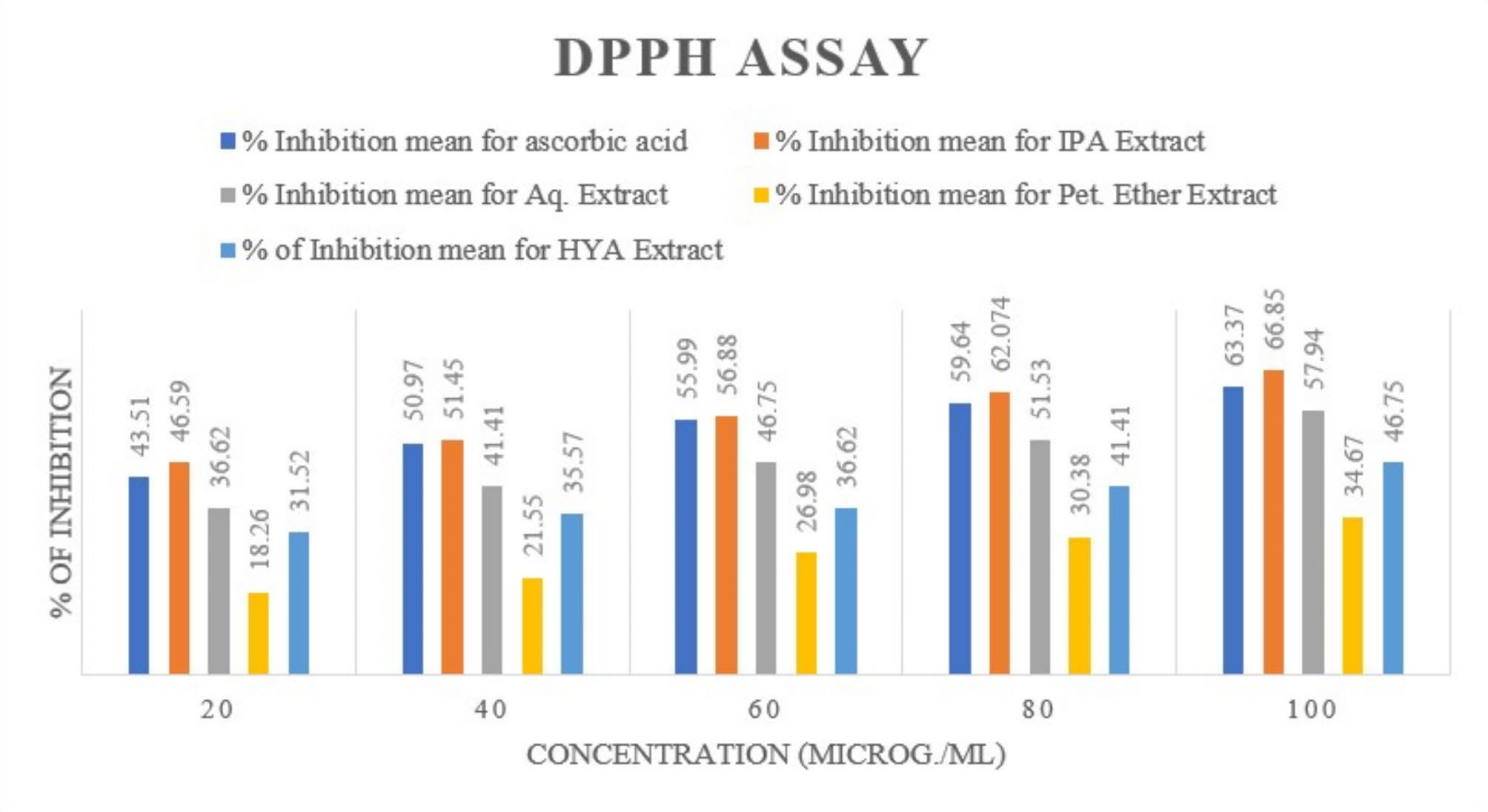
Comparison of % of inhibition of standard with the leaf extracts of *P. pudica* (Concentration in µg/mL) for DPPH assay.

A reliable and sensitive method for assessing the antioxidant activity of a specific compound or plant extract is offered by the DPPH stable free-radical technique. This method is rapid, straightforward, and does not depend solely on the type of plant but also on the extraction procedure. DPPH radical scavenging efficiency is detected at 517 nm^24^. DPPH must accept an electron or hydrogen radical to become a stable diamagnetic molecule. The reduction of DPPH radical absorbance results in a color shift from purple to yellow or greenish, indicating the presence of antioxidants in the solution that combat free radicals. The degree of inhibition is a metric for determining the antioxidant activity of the extract and its capacity to suppress free radicals. In Fig. 11, at the highest concentration (100 µg/mL), the % inhibition by standard ascorbic acid was 63.37%, and the IPA extract demonstrated the highest % inhibition at 66.85%. The IC_50_ value represents the concentration of the sample required to inhibit 50% of free radicals. A low IC_50_ value indicates a high antioxidant value^25^. In this study, the IC_50_ value of standard ascorbic acid was approximately 37.19 µg/mL, and the IPA extract had the lowest IC_50_ value at 33.54 µg/mL (Table 9).

**Table 9:** IC_50_ values of DPPH Assay for various leaf extracts of *P. pudica*.

Sample	IC_50_ (µg/mL)
Ascorbic acid	37.1996
Isopropyl alcohol	33.54
Aqueous	71.940
Petroleum ether	231.45
Hydro-alcohol	124.055

#### Results of antimicrobial assay

**Fig. 12:**
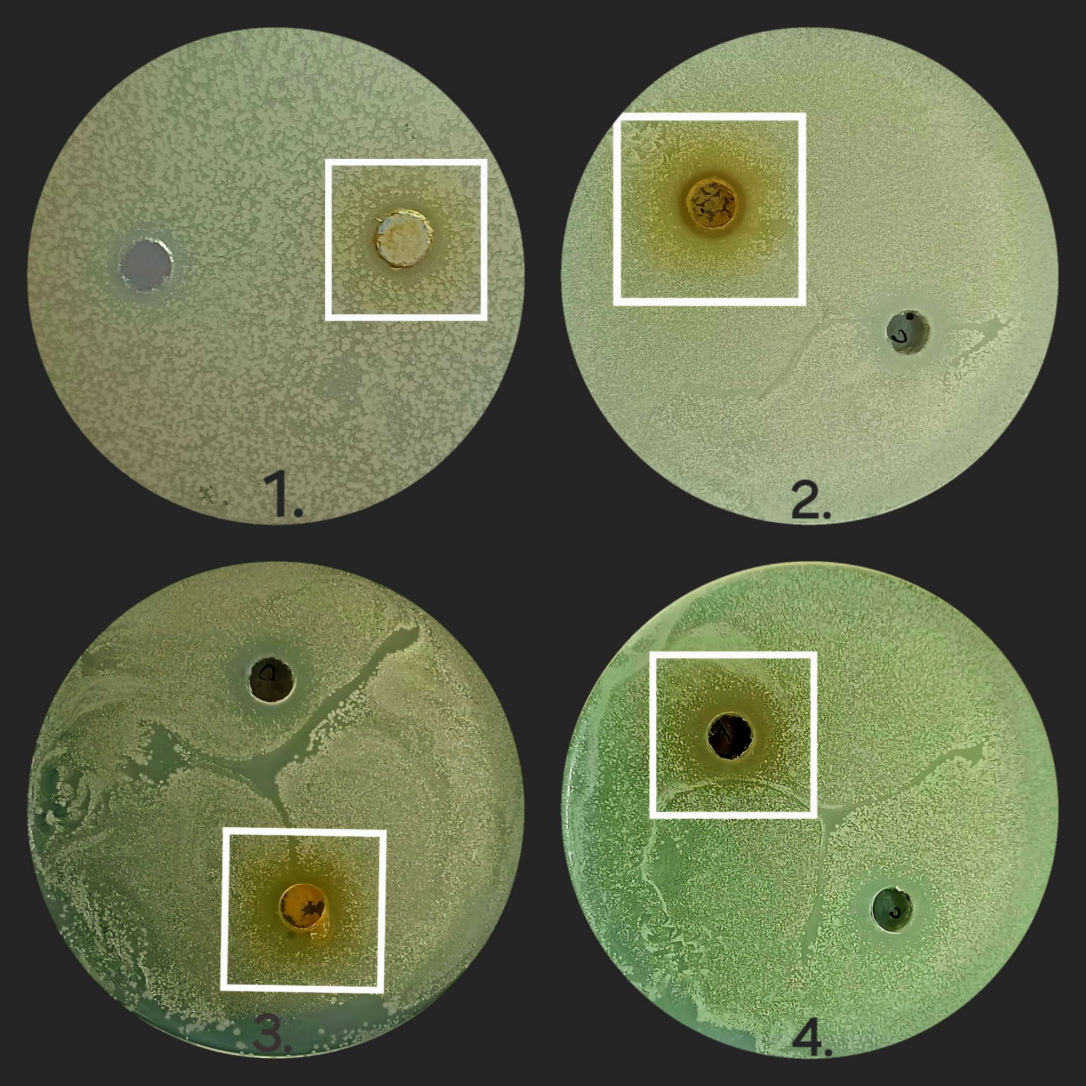
Zone of inhibition caused by leaf extracts against *Kocuria rhizophila* (Sample 1 is IPA leaf extract, 2 is Aqueous leaf extract, 3 is Petroleum ether leaf extract and 4 is Hydro alcohol leaf extract).

**Fig. 13:**
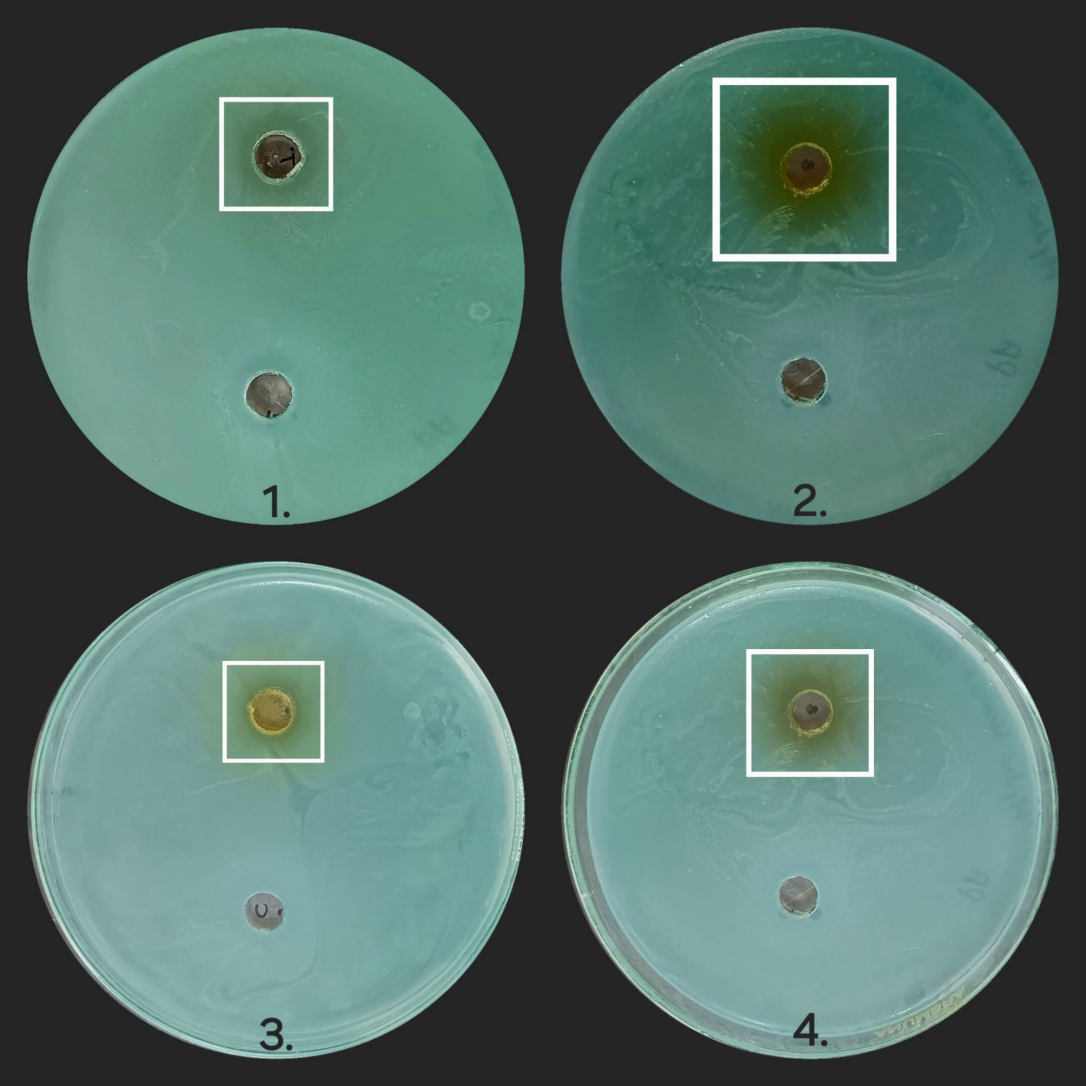
Zone of inhibition caused by leaf extracts against *Pseudomonas aeruginosa* (Sample 1 is IPA leaf extract, 2 is Aqueous leaf extract, 3 is Petroleum ether leaf extract and 4 is Hydro alcohol leaf extract).

**Fig. 14:**
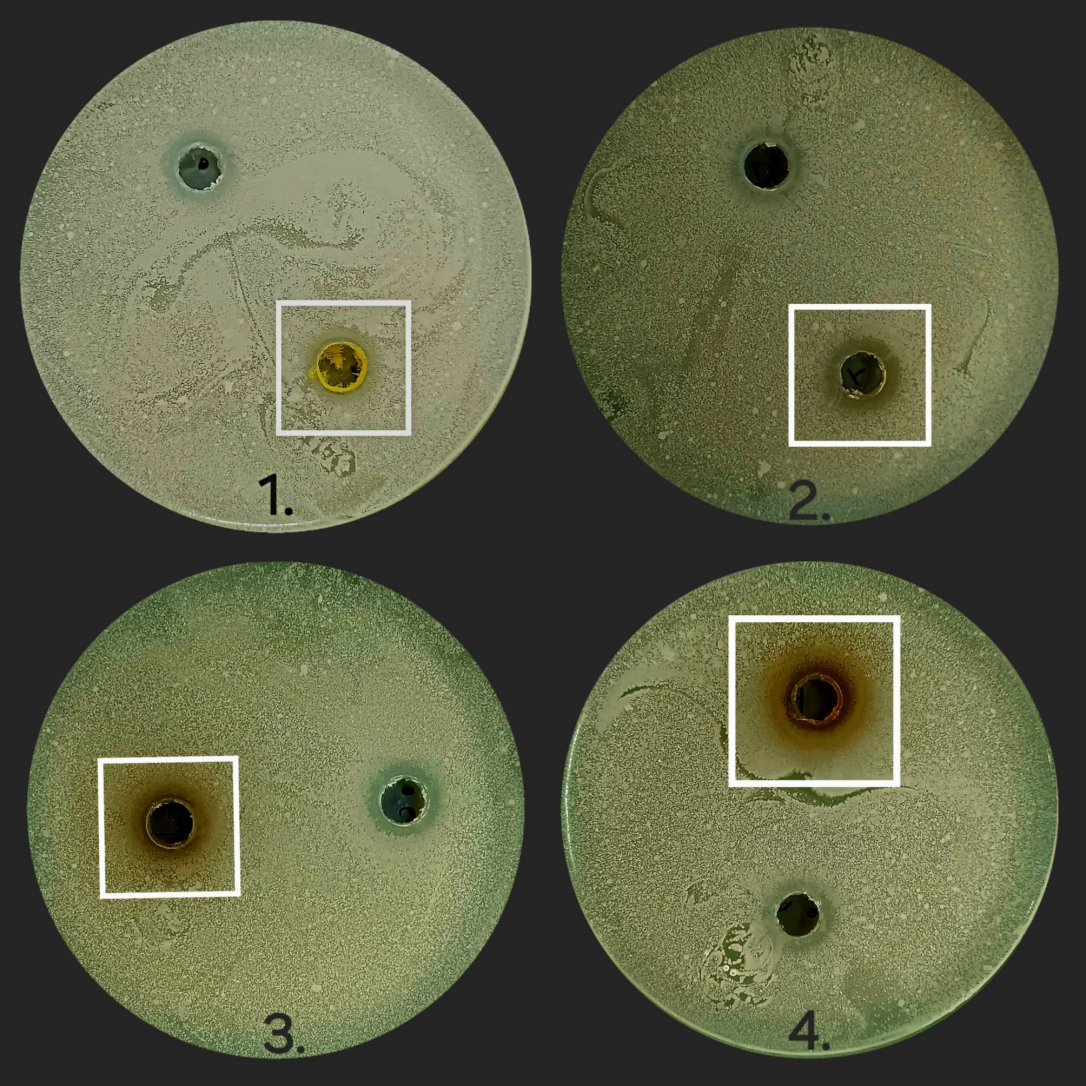
Zone of inhibition caused by leaf extracts against *Klebsiella pneumonia* (Sample 1 is IPA leaf extract, 2 is Aqueous leaf extract, 3 is Petroleum ether leaf extract and 4 is Hydro alcohol leaf extract).

**Fig. 15:**
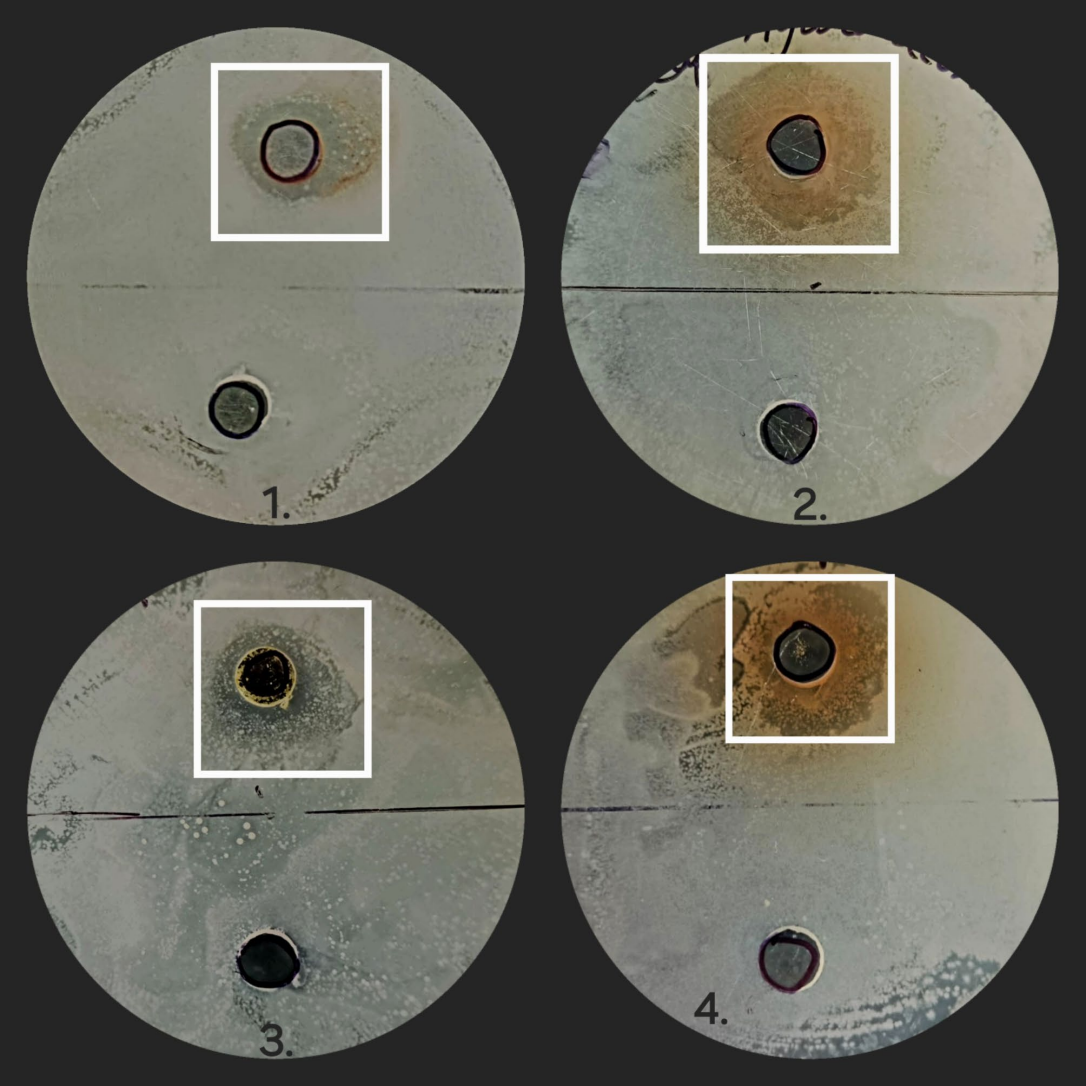
Zone of inhibition caused by leaf extracts against *Escherichia coli* (Sample 1 is IPA leaf extract, 2 is Aqueous leaf extract, 3 is Petroleum ether leaf extract and 4 is Hydro alcohol leaf extract).

**Fig. 16:**
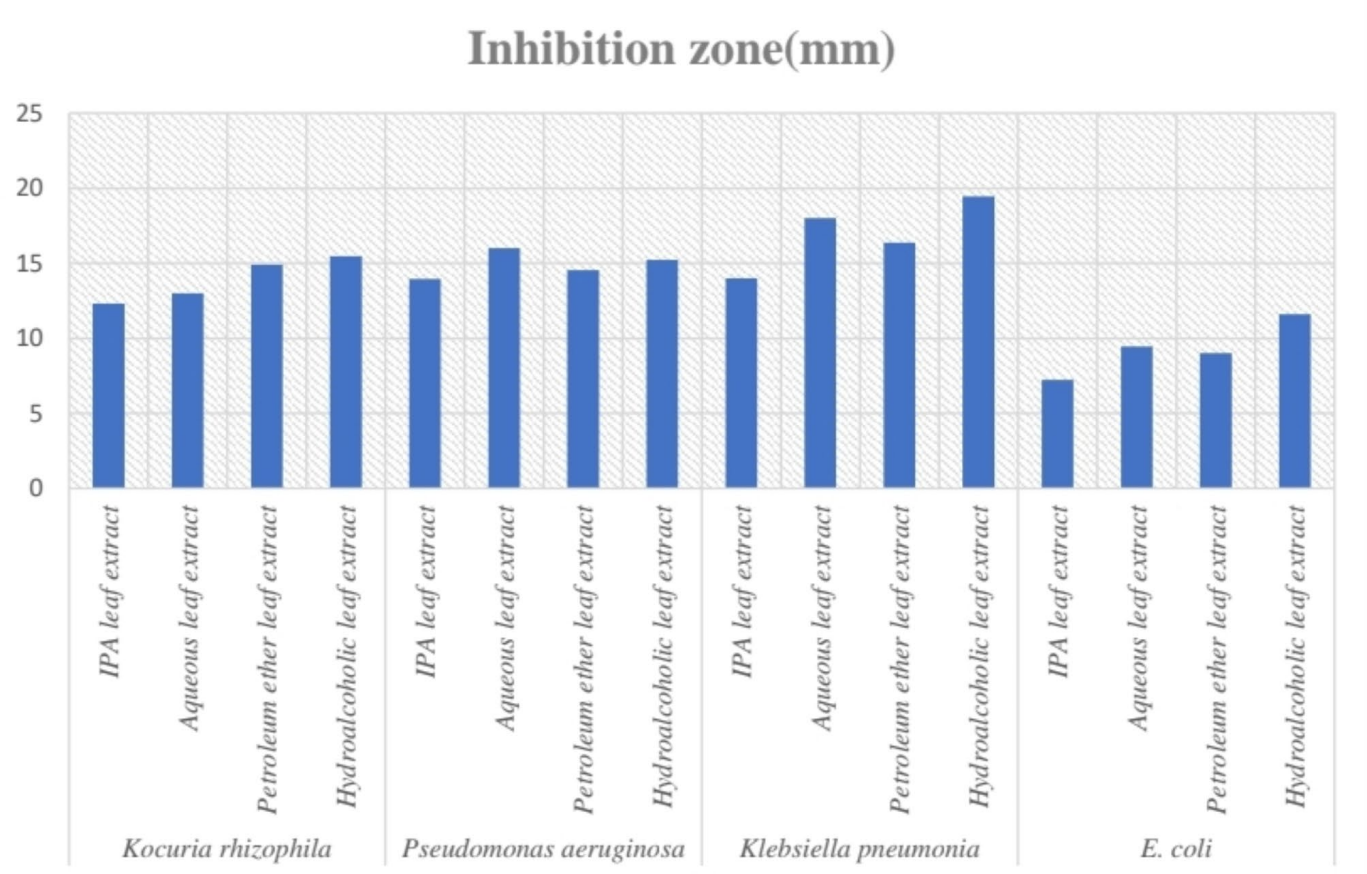
A comparative representation of the zone of inhibition demonstrated by all leaf extracts against the bacterial species.

The leaf extracts of the plant demonstrated varying degrees of antimicrobial activity (Figs. 12, 13, 14 and 15) against the tested bacteria, as shown in Table 10. The results are expressed as the zone of inhibition (ZOI) in mm. *Klebsiella pneumoniae* was the most susceptible, with the highest ZOIs: HYA leaf extract (19.45 mm), aqueous leaf extract (18 mm), petroleum ether extract (16.36 mm), and IPA leaf extract (14 mm). *E. coli* showed the greatest resistance, with the ZOIs being: HYA leaf extract (11.61 mm), aqueous leaf extract (9.45 mm), petroleum ether extract (9.02 mm), and IPA leaf extract (7.23 mm). *Pseudomonas aeruginosa* and *Kocuria rhizophila* also showed good susceptibility. For *Pseudomonas aeruginosa*, the ZOIs were: aqueous leaf extract (16 mm), HYA leaf extract (15.23 mm), petroleum ether extract (14.55 mm), and IPA leaf extract (13.95 mm). For *Kocuria rhizophila*, the ZOIs were: HYA leaf extract (15.46 mm), petroleum ether extract (14.90 mm), aqueous leaf extract (13 mm), and IPA leaf extract (12.32 mm). The diagrammatic representation of the zone of inhibitions demonstrated by the extracts against the bacterial strains is given in Fig. 16. The MIC values are presented in Table 11. *Klebsiella pneumoniae* exhibited the least resistance, with MIC values ranging from 10 mg/mL to 20 mg/mL. *E. coli* showed the highest resistance, requiring 25 mg/mL MIC for IPA, aqueous, and petroleum ether extracts, and 20 mg/mL for HYA extract. For *Kocuria rhizophila* and *Pseudomonas aeruginosa*, the MIC for all leaf extracts was 20 mg/mL.

**Table 10:** Antibacterial activity of four-leaf extracts of *Plumeria pudica* Jacq. against four strains of bacteria in terms of zone of inhibition at MIC.

Bacterial strain	Plant extract	Inhibition zone (mm)
*Kocuria rhizophila*	IPA leaf extract	12.32
	Aqueous leaf extract	13
	Petroleum ether leaf extract	14.90
	Hydroalcoholic leaf extract	15.46
*Pseudomonas aeruginosa*	IPA leaf extract	13.95
	Aqueous leaf extract	16
	Petroleum ether leaf extract	14.55
	Hydroalcoholic leaf extract	15.23
*Klebsiella pneumonia*	IPA leaf extract	14
	Aqueous leaf extract	18
	Petroleum ether leaf extract	16.36
	Hydroalcoholic leaf extract	19.45
*E. coli*	IPA leaf extract	7.23
	Aqueous leaf extract	9.45
	Petroleum ether leaf extract	9.02
	Hydroalcoholic leaf extract	11.61

**Table 11:** Expression of antimicrobial activity in terms of Minimum inhibitory concentration (MIC mg/mL) for the various leaf extracts of *P. pudica*.

Extracts	*K. rhizophila*	*P. aeruginosa*	*K. pneumonia*	*E. coli*
IPA leaf extract	20	20	20	25
Aqueous leaf extract	20	20	15	25
Petroleum ether leaf extract	20	20	20	25
Hydroalcoholic leaf extract	20	20	10	20

#### Correlations

**Fig. 17:**
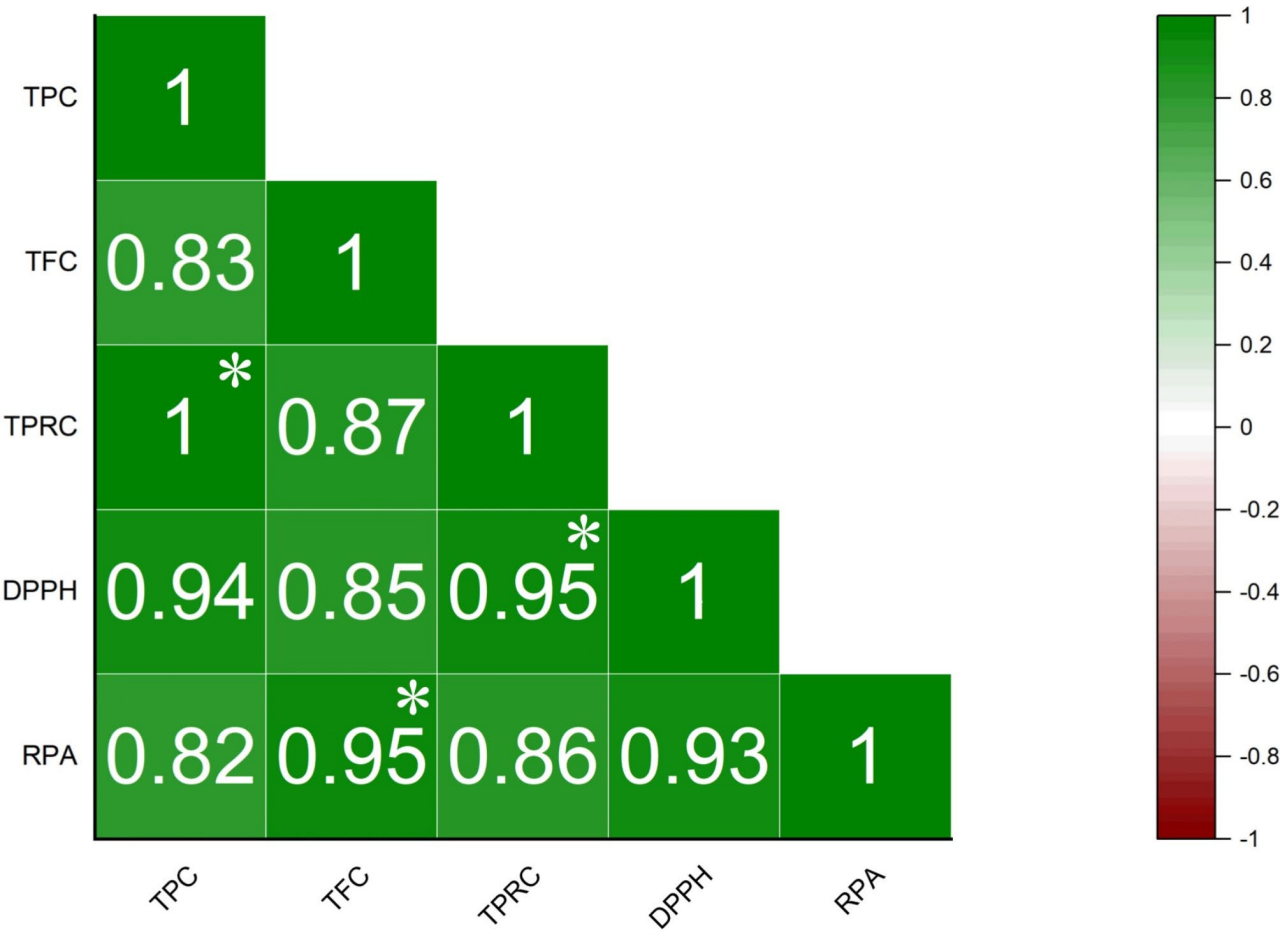
Correlation among various assays viz. TPC, TFC, TPRC, DPPH, and RPC. (*p <  = 0.05) (Here TPC = Total phenolic content, TFC = Total flavonoid content, TPRC = Total protein content, RPC = Reducing power assay).

The correlations among the results of the Total Phenolic Content, Total Flavonoid Content, Total Protein Content, DPPH, and Reducing Power assays for various extracts of *Plumeria pudica* leaves are illustrated in Fig. 17. The correlation coefficients (r) for the different content estimations and antioxidant studies ranged from 0.82 to 0.99, indicating a strong correlation across these assays. Notably, the highest correlation was observed between the Total Flavonoid Content and the Reducing Power assay (r = 0.95), while the DPPH assay showed its strongest correlation with Total Phenolic Content (r = 0.94). The lowest correlation coefficient was recorded between the Reducing Power assay and Total Protein Content (r = 0.82). The significant correlation between Total Phenolic Content and Total Flavonoid Content across various leaf extracts suggests that polyphenolic compounds and flavonoids constitute the primary phytochemicals responsible for the plant’s overall antioxidant potential. Furthermore, the Total Protein Content may also contribute to the observed antioxidant activities, as the correlation coefficients between the Total Protein Content and the DPPH and Reducing Power assays ranged from 0.82 to 0.94, which remains considerably high.

## Discussion

The medicinal significance of plants, recognized for their diverse range of pharmacologically active metabolites, has gained substantial attention in recent decades^26^. This study evaluates the impact of solvent polarity on extraction efficiency, demonstrating that polar solvents like IPA yielded the best results, while non-polar solvents like petroleum ether yielded the least. Specifically, the IPA extract of *P. pudica* showed the highest total flavonoid content (402.2 mg QE/g dry-weight of plant extract), whereas the petroleum ether extract had the lowest (65.54 mg QE/g dry-weight of plant extract). Comparatively, *P. alba* ethanolic extract had 31.26 mg QE/g dry-weight of plant the extract^27^ and *P. rubra* ethanolic extract had 117.83 mg QE/g dry-weight of plant the extract^28^, aligning closely with the TFC of *P. pudica*. Protein content was highest in the IPA extract (164 mg BSAE/g dry-weight of plant extract) and lowest in the hydroalcoholic extract (115.8 mg BSAE/g dry-weight of plant extract). The IPA extract also had the highest total phenolic content (101 mg GAE/g dry-weight of plant extract), compared to the petroleum ether extract (50.8 mg GAE/g dry-weight of plant extract). Attasih et al.^27^ reported 48.43 mg GAE/g dry-weight of plant extract in *P. alba*, which is lower than in *P. pudica*. The IPA extract had the highest carbohydrate content (336 mg GLUE/g dry-weight of plant extract), while the petroleum ether extract had the lowest (153.07 mg GLUE/g dry-weight of plant extract). The hydroalcoholic extract had the highest saponin content (202 mg DIE/g dry-weight of plant extract), and the aqueous extract had the lowest (108.41 mg DIE/g dry-weight of plant extract), with petroleum ether lacking saponins. These findings support the hypothesis that solvent polarity affects the extraction of phytoconstituents. These above qualitative and quantitative studies for phytoconstituents hence proved the hypothesis for the effect of choosing polar or non-polar solvents in terms of extraction potential which will eventually affect the further biochemical studies that are going to be conducted.

Free radicals, generated through various processes, can induce oxidative stress and contribute to diseases such as cancer and Parkinson’s disease^29–32^. Phenolic compounds are recognized for their ability to neutralize free radicals. The antioxidant potential of phenolics is primarily attributed to their redox properties, enabling them to function as reducing agents and hydrogen donors^5^. Research indicates that phenolics play a crucial preventive role in the onset of conditions such as cancer, heart disease, and age-related ailments. Flavonoids serve as protective agents for plants against various biotic and abiotic stresses, functioning as unique UV filters, signal molecules, allopatric compounds, phytoalexins, and detoxifying agents^33^. The Antioxidant potential was assessed using the DPPH assay and the reducing power assay. At 100 µg/mL, ascorbic acid exhibited 63.37% inhibition, while IPA extract showed the highest inhibition at 66.85%. The IPA extract surpassed the ascorbic acid standard in inhibition percentage. Comparable studies^31,33^ show varied inhibition percentages for other Plumeria species, indicating that *P. pudica* exhibits notable antioxidant potential. The IC_50_ value for ascorbic acid was 37.1996 µg/mL, with the IPA extract showing the lowest IC_50_ value (33.54 µg/mL), indicating superior antioxidant activity. Muhtadi and Wiyono^28^ found higher IC_50_ values in *P. rubra* and *P. alba*, suggesting *P. pudica* has better antioxidant properties. The reducing power assay corroborated these findings, with the IPA extract showing the highest absorbance (1.53) at 1000 µg/mL, compared to ascorbic acid (1.19) and petroleum ether extract (0.55). With these assays, we can conclude that there is a correlation between the presence of phenolics and flavonoids and their attribution to the antioxidant potential of the extract as the IPA leaf extract had the highest TPC (101 mg GAE/g dry-weight of plant extract) and the highest TFC (402.2 QE/g of dry-weight of plant extract), thus it showed best results in both reducing power assay and DPPH assay.

Antimicrobial studies revealed *Klebsiella pneumoniae* as highly susceptible to the leaf extracts, with ZOIs from 19.45 mm (HYA extract) to 14 mm (IPA extract). *E. coli* showed the highest resistance, with ZOIs from 11.61 mm (HYA extract) to 7.63 mm (IPA extract). Despite its lesser-known status, *P. pudica* holds significant potential for developing plant-based drugs. Further comprehensive studies are necessary to explore its anti-cancer, anti-inflammatory, and anti-nociceptive properties and its broader therapeutic potential.

## Conclusion

The principal objective of this study is to identify the presence of different secondary metabolites, and the quantity of five of the phytoconstituents, and to evaluate the anti-oxidant and antimicrobial potential of the four-leaf extracts of the plant *Plumeria pudica*. The overall presence of various phytoconstituents sheds light on the pharmaceutical potential of the plant. The Isopropyl leaf extract of the plant showed the best results in terms of the qualities and quantities of the phytochemicals and also, the high phenolic content present in leaves favors high anti-oxidant activity. Also, both the DPPH and reducing power assays showed excellent results thus proving the plants’ pharmacological potential. The antimicrobial evaluation showcased the plant’s potential as a medicinal remedy for the treatment of various diseases. Additionally, the isolation, purification, and characterization of the phytochemicals will simplify leading further studies on the discovery of bioactive compounds, resolving their efficacy by in vivo studies, and demonstrating their safety and effectiveness in clinical trials.

## Data Availability

All the data that has been generated during this research are included within the manuscript.

## Abbreviations

IPA: Iso-propyl alcohol
HYA: Hydro alcohol
DPPH: 1,1-diphenyl-2-picrylhydrazyl
FC: Folin–Ciocalteau
GAE: Gallic acid equivalent
QE: Quercetin equivalent
BSAE: Bovine serum albumin equivalent
GLE: Glucose equivalent
DIE: Diosgenin equivalent
TPC: Total phenol content
TFC: Total flavonoid content

## References

[1] Mann, J. Secondary Metabolism: Oxford Chemistry Series (1978).

[2] Akbulut, H. F. & Akbulut, M. Mineral composition, the profile of phenolic compounds, organic acids, sugar, and in vitro antioxidant capacity, and antimicrobial activity of organic extracts of Juniperus drupacea fruits. *Food Sci. Nutr.* **11**, 6435–6446 (2023). 37823141 10.1002/fsn3.3586 PMC10563755

[3] Chamakuri, S. R., Suttee, A. & Mondal, P. An eye-catching and comprehensive review of *Plumeria pudica* Jacq. (Bridal Bouquet). *Plant Arch*. **20**(2), 2076–2079.

[4] Radhika, B. Pharmacogenetic evaluation of the leaves of *Plumeria pudica*. *J. Nat. Prod. Plant Res.* **7**, 40–45 (2020).

[5] Santana, L. D. A. B. et al. Antidiarrheal effects of water-soluble proteins from *Plumeria pudica* latex in mice. *Biomed. Pharmacother.* **97**, 1147–1154 (2018). 29136953 10.1016/j.biopha.2017.11.019

[6] Larson, R. A. The antioxidants of higher plants. *Phytochemistry* **27**(4), 969–978. 10.1016/0031-9422(88)80254-1 (1988).

[7] Fernandes, H. B. et al. Laticifer proteins from *Plumeria pudica* inhibit the inflammatory and nociceptive responses by decreasing the action of inflammatory mediators and pro-inflammatory cytokines. *Rev. Bras. Farmacogn.* **25**, 269–277 (2015).

[8] Arya, V., Thakur, N. & Kashyap, C. P. Preliminary phytochemical analysis of the extracts of *Psidium* leaves. *J. Pharmacogn. Phytochem.* **1**, 01–05 (2012).

[9] Patel, P., Patel, N., Patel, D., Desai, S. & Meshram, D. Phytochemical analysis and antifungal activity of *Moringa oleifera*. *Int. J. Pharm. Pharm. Sci.* **6**(5), 144–147 (2014).

[10] Singelton, V. L. Lamuela-Raventos: Analysis of total phenols and other oxidation substrates and antioxidants by means of Folin-Ciocalteu reagent. *Methods Enzymol.* **299**, 152 (1999).

[11] Lowry, O. H., Rosebraugh, N. J., Farr, A. L. & Randall, R. J. Protein measurement with the Folin-Phenol reagent. *J. Biol. Chem.* **193**, 265–275 (1951). 14907713

[12] Tamboli, F. A., More, H. N., Bhandugare, S. S., Patil, A. S., Jadhav, N. R. & Killedar, S. G. Estimation of total carbohydrate content by phenol sulphuric acid method from *Eichhornia crassipes* (Mart.). Solms (2020).

[13] Mokrani, A. et al. Phenolic contents and bioactive potential of peach fruit extracts. *Food Chem.* **202**, 212–220. 10.1016/j.foodchem.2015.12.026 (2020). 10.1016/j.foodchem.2015.12.026 26920287

[14] Aiyengar, O. A. & Okoh, A. I. Preliminary phytochemical screening and in vitro antioxidant activities of the aqueous extract of *Helichrysum longifolium* DC. *BMC Complement. Altern. Med.* **10**(1), 1–8. 10.1186/1472-6882-10-21 (2010). 20470421 10.1186/1472-6882-10-21 PMC2877649

[15] Kupe, M. et al. Phenolic composition and antioxidant activity of peel, pulp, and seed extracts of different clones of the Turkish grape cultivar ‘Karaerik’. *Plants* **10**, 2154 (2021). 34685965 10.3390/plants10102154 PMC8538078

[16] Hiai, S., Oura, H. & Nakajima, T. Color reaction of some sapogenins and saponins with vanillin and sulfur1c acid. *Planta Med.* **29**(02), 116–122 (1976). 948509 10.1055/s-0028-1097639

[17] Nithianantham, K. et al. Hepatoprotective potential of *Clitoria ternate* leaf extract against paracetamol-induced damage in mice. *Molecules* **16**(12), 10134–10145. 10.3390/molecules161210134 (2011). 22146374 10.3390/molecules161210134 PMC6264671

[18] Brand-Williams, W., Cuvelier, M. E. & Berset, C. L. W. T. Use of a free radical method to evaluate antioxidant activity. *LWT Food Sci. Technol.* **28**, 25–30. 10.1016/S0023-6438(95)80008-5 (1995).

[19] Stankovic, M. S. Total phenolic content, flavonoid concentration, and antioxidant activity of *Marrubium peregrinum* L. extracts. *Kragujevac J. Sci.* **33**, 63–72 (2011).

[20] Wald, M., Schwarz, K., Rehbein, H., Bußmann, B. & Beermann, C. Detection of antibacterial activity of an enzymatic hydrolysate generated by processing rainbow trout by-products with trout pepsin. *Food Chem.* **205**, 221–228 (2016). 27006234 10.1016/j.foodchem.2016.03.002

[21] Sa-Eed, A. et al. In vitro antimicrobial activity of crude propolis extracts and fractions. *FEMS Microbes* **4**, xtad010 (2023). 37333437 10.1093/femsmc/xtad010 PMC10165684

[22] Performance CLSI. Standards for antimicrobial susceptibility testing, CLSI supplement M100S (Clinical and Laboratory Standards Institute, 2016).

[23] Wiegand, I., Hilpert, K. & Hancock, R. E. Agar, and broth dilution methods to determine the minimal inhibitory concentration (MIC) of antimicrobial substances. *Nat. Protoc.* **3**, 163–175 (2008). 18274517 10.1038/nprot.2007.521

[24] Jadid, N., Hidayati, D., Hartanti, S. R., Arraniry, B. A., Rachman, R. Y. & Wikanta, W. Antioxidant activities of different solvent extracts of *Piper retrofractum* Vahl. using DPPH assay. In *AIP Conference Proceedings* Vol. 1854 (AIP Publishing, 2017). 10.1063/1.4985410.

[25] Wrasiati, L. P., Wirawan, I. G. P., Bagiada, N. A. & Astawa, I. N. M. Antioxidant capacity of frangipani (*Plumeria alba*) powder extract. *Indones. J. Biomed. Sci.* **5** (2011).

[26] Cavalcanti, R. et al. Natural product extraction. *Plant Antioxidants and Health.*10.1039/9781849737579 (2011).

[27] Attasih, M., Pambudi, D. B. & Saad, M. Determination of total phenolic, flavonoid contents, and antioxidant activity evaluation of ethanolic extract from *Plumeria alba*. *J. Nutraceuticals Herb. Med.* 14–27 (2024).

[28] Muhtadi, M. & Wiyono, A. A. F. Testing antioxidant activity of *Plumeria alba* and *Plumeria rubra* ethanolic extracts using DPPH and Frap methods and determining their total flavonoid and phenolic levels. *J. Nutraceuticals Herb. Med.* **3**, 38–50 (2021).

[29] Liu, W. & Speranza, G. Functionalization of carbon nanomaterials for biomedical applications. *C J. Carbon Res.* **5**, 72. 10.3390/c5040072 (2019).

[30] Gülçin, İ. The antioxidant and radical scavenging activities of black pepper (*Piper nigrum*) seeds. *Int. J. Food Sci. Nutr.* **56**, 491–499. 10.1080/09637480500450248 (2005). 16503560 10.1080/09637480500450248

[31] Hsu, C. L., Chen, W., Weng, Y. M. & Tseng, C. Y. Chemical composition, physical properties, and antioxidant activities of yam flours as affected by different drying methods. *Food Chem.* **83**, 85–92. 10.1016/S0308-8146(03)00053-0 (2003).

[32] Rahman, T., Hosen, I., Islam, M. M. T. & Shekhar, H. U. Oxidative stress and human health. *Adv. Biosci. Biotechnol.* **3**, 997–1019. 10.4236/abb.2012.327123 (2012).

[33] Samanta, A., Das, G. & Das, S. K. Roles of flavonoids in plants. *Carbon* **100**, 12–35 (2011).

